# Utilizing field collected insects for next generation sequencing: Effects of sampling, storage, and DNA extraction methods

**DOI:** 10.1002/ece3.5756

**Published:** 2019-12-03

**Authors:** Kimberly M. Ballare, Nathaniel S. Pope, Antonio R. Castilla, Sarah Cusser, Richard P. Metz, Shalene Jha

**Affiliations:** ^1^ Department of Integrative Biology The University of Texas at Austin Austin TX USA; ^2^ Genomics and Bioinformatics Service Texas A&M AgriLife Research College Station TX USA; ^3^ Present address: Department of Ecology and Evolutionary Biology University of California Santa Cruz Santa Cruz CA USA; ^4^ Present address: Department of Entomology Pennsylvania State University University Park PA USA; ^5^ Present address: Centre for Applied Ecology “Prof. Baeta Neves”/INBIO Instituto Superior of Agronomy University of Lisbon Lisbon Portugal; ^6^ Present address: Kellogg Biological Station Michigan State University Hickory Corners MI USA

**Keywords:** curated insects, ddRAD, degraded DNA, next generation sequencing, pan traps, propylene glycol

## Abstract

DNA sequencing technologies continue to advance the biological sciences, expanding opportunities for genomic studies of non‐model organisms for basic and applied questions. Despite these opportunities, many next generation sequencing protocols have been developed assuming a substantial quantity of high molecular weight DNA (>100 ng), which can be difficult to obtain for many study systems. In particular, the ability to sequence field‐collected specimens that exhibit varying levels of DNA degradation remains largely unexplored. In this study we investigate the influence of five traditional insect capture and curation methods on Double‐Digest Restriction Enzyme Associated DNA (ddRAD) sequencing success for three wild bee species. We sequenced a total of 105 specimens (between 7–13 specimens per species and treatment). We additionally investigated how different DNA quality metrics (including pre‐sequence concentration and contamination) predicted downstream sequencing success, and also compared two DNA extraction methods. We report successful library preparation for all specimens, with all treatments and extraction methods producing enough highly reliable loci for population genetic analyses. Although results varied between species, we found that specimens collected by net sampling directly into 100% EtOH, or by passive trapping followed by 100% EtOH storage before pinning tended to produce higher quality ddRAD assemblies, likely as a result of rapid specimen desiccation. Surprisingly, we found that specimens preserved in propylene glycol during field sampling exhibited lower‐quality assemblies. We provide recommendations for each treatment, extraction method, and DNA quality assessment, and further encourage researchers to consider utilizing a wider variety of specimens for genomic analyses.

## Graphical abstract

In this study, we compare effects of capture method, preservation method, and DNA extraction method on next‐generation sequencing success for three different bee species using ddRAD sequencing. Our results indicate that specimens that were hand‐netted into and stored in EtOH, and those that were trapped and pinned exhibited high‐quality ddRAD results, but, surprisingly, we found that specimens trapped using propylene glycol, a method assumed to preserve DNA, performed poorly. We additionally found little difference between the higher and low‐cost DNA extraction methods, and describe recommendations for use of DNA quality metrics that are predictive of downstream sequencing and assembly success.

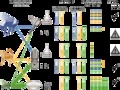

## 1 INTRODUCTION

The rapid expansion of molecular techniques throughout the last 60 years has revolutionized the field of biology and the study of wild populations across taxonomic and spatial scales. Development of various molecular markers and sequencing technologies have allowed for essential empirical tests of population genetics theory (Charlesworth & Charlesworth, 2017), improved resolution of phylogenies (Whelan, Liò, & Goldman, 2001), greater insight into behavioral and evolutionary ecology (Hughes, 1998; Woodard et al., 2015), and a more informed identification of genetic conservation units (DeSalle & Amato, 2004). Most recently, next generation sequencing (NGS) techniques have been employed to advance understanding of critical ecological and evolutionary processes for non‐model species (Davey et al., 2011; Woodard et al., 2015). Specifically, the increased genome coverage provided by NGS markers and the recent advancement of assembly pipelines for non‐model species have made NGS techniques especially suitable for the investigation of species where sample availability may be limited or where sample quality may be degraded. For example, one recent study used NGS techniques (shotgun sequencing) to compare museum specimens from an extinct mainland population of the Lord Howe Island stick insect, an extremely rare and evolutionarily distinct species, with a newly discovered extant island morph. These data confirmed that the two populations belong to the same species, providing evidence that the island individuals may be suitable for mainland reintroduction (Mikheyev et al., 2017). Given the large number of species that are ecologically critical yet understudied (e.g. Fisher, Knowlton, Brainard, & Caley, 2011; McKinney, 1999), and the increasing interest in the evolutionary ecology of non‐model organisms (Ekblom & Galindo, 2011), NGS tools provide key opportunities for the exploration of basic biological questions and the development of species‐specific conservation guidelines.

However, despite the great potential of NGS tools, many protocols have been developed assuming that a substantial quantity of high molecular weight DNA should be used for library preparation. For example, in a recent review of NGS methods, approximately 80% of the studies cited used tissue that was either freshly sampled or preserved in EtOH (Andrews, Good, Miller, Luikart, & Hohenlohe, 2016). This pattern belies the difficulty of obtaining high quality DNA for many species, the greater availability of specimens that may have lower quality DNA, and the untapped potential of these samples to address key questions in ecology and evolution. Tremendous biological insight can be gained from specimens which may have degraded DNA due to environmental or storage conditions including road‐killed specimens (Rusterholz, Ursenbacher, Coray, Weibel, & Baur, 2015; Say, Devillard, Léger, Pontier, & Ruette, 2012), shed substances including feces, feathers, or fur (Alda et al., 2013; Hans et al., 2015; Waits & Paetkau, 2005), as well as museum and herbarium specimens (Beck & Semple, 2015; Gilbert, Moore, Melchior, & Worobey, 2007; Sproul & Maddison, 2017). Recent work on ancient specimens has revealed great potential for NGS with very limited amounts of highly degraded DNA (Heintzman et al., 2015; Knapp & Hofreiter, 2010; Kosintsev et al., 2018), although most ancient studies focus on large vertebrate taxa (but see Heintzman, Elias, Moore, Paszkiewicz, & Barnes, 2014). Thus, there is a need for better understanding more taxonomically diverse groups, like insects, which represent an estimated 40% of the world's non‐microbial biodiversity (Scheffers, Joppa, Pimm, & Laurance, 2012), but have limited marker‐based resources and typically need to be dried and pinned before expert identification is possible (Wheeler & Miller, 2017), possibly leading to greater DNA degradation than in other taxa.

**Figure 1:**
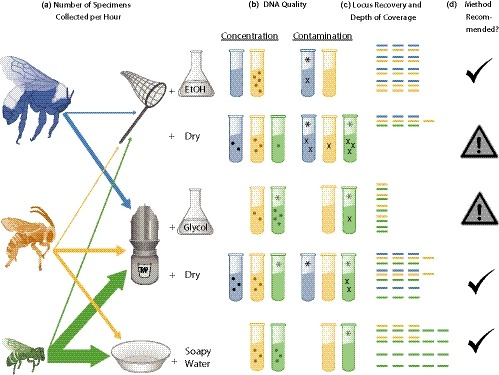
Summary of methods and results including (a) Mean number of specimens caught per active researcher hour, (b) DNA quality, (c) Locus Recovery & Depth of Coverage, and (d) Recommendation of each method for ddRAD analyses. (a) Arrows are weighted by mean number of specimens per species (*Bombus pensylvanicus* (blue); *Melissodes tepaneca* (gold); *Lasioglossum bardum* (green)) captured per sampling method (1 font size = 1 bee caught per active hour; this was calculated from all specimens of these species in our collection (*n* = 737)). The field sampling methods include (top to bottom): hand netting into either EtOH (sample code “Net‐EtOH”) or an ethyl acetate kill jar (“Net‐Dry”), blue vane trapping into either propylene glycol (“Vane‐Gly”) or no preservative (“Vane‐Dry”), and pan trapping into soapy water (“Pan”). (b) Mean Concentration per treatment per species indicated by dots per tube, with one dot per one ng/µl post‐cleanup DNA concentration above 10 ng/µl (i.e. 1 dot = 11 ng/µl, 2 dots = 12 ng/µl, etc), and Mean Contamination is indicated by black X's per tube, with one X per 0.30 value below 2.0, where a value of 2.0–2.2 has no X's and indicates “pure” DNA in the NanoDrop 260/230 index (the results for the 260/280 index are not displayed). (c) Because locus recovery and depth of coverage varied dramatically between species, differences are represented as standard deviations of the mean per species. Thus, each column represents Locus Recovery where the mean number of loci within a species is represented by three columns (measured as the logit transformed mean probability of loci occurring in another sample), plus or minus one bar for each 1/2 standard deviation away from the mean. Likewise, number of rows represents Mean Depth of Coverage where three rows is equal to the mean log transformed locus depth per species, plus or minus one bar for each 1/2 standard deviation away from the mean. For example, *B. pensylvanicus* specimens (blue bars) sampled with the Net‐EtOH treatment showed average levels of locus recovery among all treatments for *B. pensylvanicus* (three columns) but higher‐than‐average depth of coverage among all treatments for *B. pensylvanicus* (four rows). In contrast, *M. tepaneca* specimens (yellow bars) sampled with the Vane‐Dry treatment showed higher‐than average levels of locus recovery (four columns), but lower‐than‐average depth (two rows). (d) Recommendations for use of method are based on active researcher time collecting specimens, DNA quality, and locus recovery & depth of coverage results for all species, where highly recommended methods have a black checkmark and lower quality methods (to be used with caution) are denoted with a caution symbol. All methods produced ddRAD data, but Net‐Dry and Vane‐Gly specimens are not highly recommended due to various negative factors summarized in columns a–c. *Asterisks indicate significant (*p* < .05) or marginally significant (*p* < .10) differences between treatments within a species for DNA quality. See text for significant differences between treatments for locus recovery and depth

Specifically, molecular studies of insects can be challenging because many of the standard field sampling and lab preservation methods that are traditionally used in entomology collections are especially prone to DNA degradation problems. The most common ways of collecting insects often involve capturing insects in traps without preservative, or in soapy water, where they can remain for several days (e.g. pan, pitfall, and blue vane traps; LeBuhn, Griswold, Minckley, & Droege, 2003; Potts et al., 2005; Rubink, Murray, & Baum, 2003), likely leading to DNA degradation. Hand netting into a kill jar may expose specimens to chemicals that could also degrade DNA (e.g. ethyl acetate, Dillon, Austin, & Bartowsky, 1996). Trapping using a preservative such as propylene glycol has been shown to be an effective method for DNA preservation of several invertebrate species (Dillon et al., 1996; Ferro & Park, 2013), but the effects of propylene glycol on NGS results are not known. Although trapping into an EtOH preservative could potentially be done, EtOH evaporates rapidly and so it must be refilled, which can be a hinderance to sampling in high temperatures, over large geographic scales, or longer time periods; in contrast, trapping without preservative in the trap or with propylene glycol is common in low resource areas (e.g., desert and dry grasslands) as these traps may be set for several days or even months at a time (Rubink et al., 2003; Stephen & Rao, 2005; Sudan, 2016). While some studies suggest netting samples directly into EtOH for NGS (e.g. Moreau, Wray, Czekanski‐Moir, & Rubin, 2013), this technique also presents major challenges as it is time‐consuming, with a capture rate that can be 3‐17x lower than trapping (Figure 1; Roulston, Smith, & Brewster, 2007; Stephen & Rao, 2007), and can only be used on field‐identifiable species. In practice, species identification of many insect groups requires morphological trait assessment that can only be determined under magnification and after pinning and drying in order to visualize minute morphological features (Huber, 1998), some of which are compromised after long‐term storage in EtOH (e.g. hair color and growth patterns; J. Neff, pers. comm.). However, specimen curation that forgoes EtOH storage and instead optimizes morphological identification and specimen cataloguing may lead to additional DNA degradation (Andersen & Mills, 2012; Gilbert, Moore, Melchior, & Worobey, 2007; Strange, Knoblett, & Griswold, 2009), that may compromise use in genetic or genomic studies.

Small DNA fragments do not automatically hinder many NGS applications, such as shotgun sequencing, as most applications are designed to sequence short fragments. Next generation sequencing has allowed protocols to be developed for sequencing highly degraded samples, such as environmental or ancient DNA (Enk et al., 2014; Knapp & Hofreiter, 2010; Thomsen & Willerslev, 2015). However, an increasingly popular NGS application for both molecular ecologists and non‐specialist laboratories is the use of reduced representation DNA libraries (RRLs) which use restriction enzyme digestion of the genome allowing for the creation of relatively low‐cost libraries for SNP discovery and genotyping across multiplexed samples (Davey et al., 2011). Because the RRL approach relies upon analyzing restriction enzyme cut fragments, it is possibly well‐suited to analyze already‐fragmented DNA, although different applications have variable tolerances to DNA degradation. One of the most popular techniques for the creation of RRLs is Double Digest Restriction Enzyme Associated DNA sequencing (ddRAD, Peterson, Weber, Kay, Fisher, & Hoekstra, 2012). The ddRAD method is often used in marker discovery (Jansson et al., 2016), whole genome association mapping (Barría et al., 2018; Wu et al., 2016), phylogenetics (DaCosta & Sorenson, 2016), and population genomics (Andrews et al., 2016; Agudelo et al., 2015). It is considered especially useful for non‐model organisms as it does not require a reference genome, though its utility for degraded specimens is still debated. For example, ddRAD requires completely intact 5′ and 3′ overhangs, and so its use may be compromised if genomic DNA quality is very low (Puritz et al., 2014). One past ddRAD study using whitefish specimens found that proportions of low‐quality reads increased exponentially for specimens with high levels of degradation (extracted 96 hr post‐euthanasia), while specimens with moderate degradation (12–48 hr post‐euthanasia) produced similar results to the non‐degraded DNA, including high levels of depth and numbers of polymorphic loci (Graham et al., 2015). Other studies have shown that degraded DNA from museum specimens (collected 50–100 years ago) can be successfully used for RAD procedures (Haponski, Lee, & Foighil,^2017^; Sproul & Maddison, 2017; Tin, Economo, & Mikheyev, 2014), but these studies have not examined the impacts of differing sampling techniques (e.g. netting vs. trapping), different DNA extraction methods, or use of different focal species on ddRAD sequencing success.

In this study we investigate the effects of capture method, preservation method, and DNA extraction method on ddRAD sequencing success for three wild bee species: *Bombus pensylvanicus*, *Melissodes tepaneca,* and *Lasioglossum bardum*. These species vary in size (large, medium, and small respectively), and are all common throughout much of North America. We assessed three main measures as proxies for evaluating ddRAD sequencing success: number of polymorphic loci, sequencing read depth, and levels of missing loci between treatments within a species. While number of loci is often utilized as a standard measurement of sequencing success (Graham et al., 2015), depth of coverage (number of sequence reads for a given locus) is also an important metric of sequence quality, as higher depth allows for greater detection of sequencing errors, heterozygous loci, and differences between individuals and populations (Maroso et al., 2018; Sims, Sudbery, Ilott, Heger, & Ponting, 2014). Quantifying the amount of missing data is also an important aspect of assessing RAD success, as there is a finite number of sequencing reads spread across multiple individuals during sequencing, and the assembly of genomes, either de novo or mapped to a reference, depends on sequence similarity (low levels of missing data) between specimens (Catchen, Amores, & Hohenlohe, 2011). Overall, we predicted that different sampling and extraction treatments would affect both DNA quality and sequencing success for the three species. Specifically, we expected that specimens that were netted directly into and stored in EtOH would have the highest levels of DNA quality and sequencing success including higher numbers of loci, higher locus depth, and less missing data among specimens. We also predicted that netted specimens which were frozen before pinning would perform better than trapped specimens. Lastly, we expected that trapped specimens which included propylene glycol as a preservative would have higher DNA quality and better sequencing results than those specimens trapped without a preservative.

## 2 MATERIALS AND METHODS

### 2.1 Insect sampling and storage

Specimens were captured using three standardized methods including hand netting, blue vane trapping (Stephen & Rao, 2007), and pan trapping (Roulston et al., 2007) at 39 sites across Texas during the summers of 2012–2014 (Table S1). Samples were collected for various projects that were primarily focused on sampling the entire bee community to answer questions related to community assembly and meta‐population analyses without considering genetic preservation consequences (Ballare, Neff, Ruppel, & Jha, 2019; Cusser, Neff, & Jha, 2016; Ritchie, Ruppel, & Jha, 2016), as is likely the case for many entomology collections (J. Neff, A. Wild, pers. comm.). Sample numbers ranged from 7 to 13 specimens per treatment based on sample availability in our collection; this sample size is higher than most methods studies that test NGS protocols, which often only include one or two specimens per treatment (Heintzman et al., 2014; Graham et al., 2015; Sproul & Maddison, 2017; but see Vaudo, Fritz, & Lopez‐Uribe, 2018). Hand‐netted specimens were collected via two methods: either directly into 100% EtOH (hereafter “Net‐EtOH”) or into a jar using ethyl acetate vapor as a killing agent (hereafter “Net‐Dry”). Blue vane‐trapped specimens were also trapped via two methods: with no preservation agent or bait in the trap (hereafter “Vane‐Dry”) or using propylene glycol as a preservation agent (hereafter “Vane‐Gly”). Propylene glycol is commonly used for short‐ and long‐term trapping (in the latter, specimens may remain in traps for several months) to preserve insects for later morphological identification (Rubink et al., 2003; Sudan, 2016). In both blue vane trapping methods, traps were left for 5 days in the field before collection. Pan‐trapped specimens (hereafter referred to as “Pan”) were trapped in small plastic bowls filled with soapy water (approximately one tablespoon Dawn^®^ dishwashing liquid/ gallon water), as per LeBuhn et al. (2003). Pan traps were left in the field for 24 hr before collection. Net‐EtOH specimens were stored at room temperature in 100% EtOH until extraction. The specimens collected by the remaining four methods were pinned and dried for long‐term storage. Net‐Dry and Vane‐Dry specimens were kept on ice for ~8 hr in the field and then frozen at −20°C before pinning, while Vane‐Gly and Pan specimens were stored in 100% EtOH at room temperature until pinning. All pinned specimens were ultimately stored at room temperature in pine Cornell drawers (BioQuip item 1012AF) within closed steel Cornell University system cabinets (BioQuip item 2525) until extraction. Due to the variation in body size of each species, variable abundance of wild non‐model organisms, and various logistic restrictions, specimens were not captured in all methods across species. For example,* B. pensylvanicus* were captured via hand netting and dry blue vane trapping, but were not captured in pan traps due to their large body size or in glycol blue vane traps due to chance (see Table 1 for specimen trapping and storage summary).

**Table 1:** Summary of sampling, storage, and DNA extraction methods for *Bombus pensylvanicus*, *Melissodes tepaneca*, and *Lasioglossum bardum,* showing *N* specimens in each treatment group

Species	Treatment Code	Field sample method	Pre‐pinning storage	Long term storage	DNA extraction method(s)	*N*
*B. pensylvanicus*	Net‐EtOH	Net, EtOH	NA	EtOH	Qiagen, DNAzol	13
	Net‐Dry	Net, Ethyl Acetate Kill Jar	−20°C	Pin	Qiagen, DNAzol	10
	Vane‐Dry	Blue Vane, No Preservative	−20°C	Pin	Qiagen, DNAzol	8
*M. tepaneca*	Net‐EtOH	Net, EtOH	NA	EtOH	Qiagen	8
	Net‐Dry	Net, Ethyl Acetate Kill Jar	−20°C	Pin	Qiagen	8
	Vane‐Dry	Blue Vane, No Preservative	−20°C	Pin	Qiagen	8
	Vane‐Gly	Blue Vane, Propylene Glycol	EtOH	Pin	Qiagen	7
	Pan	Pan Trap, Soapy Water	EtOH	Pin	Qiagen	8
*L. bardum*	Net‐Dry	Net, Ethyl Acetate Kill Jar	−20°C	Pin	Qiagen	9
	Vane‐Dry	Blue Vane, No Preservative	−20°C	Pin	Qiagen	10
	Vane‐Gly	Blue Vane, Propylene Glycol	EtOH	Pin	Qiagen	8
	Pan	Pan Trap, Soapy Water	EtOH	Pin	Qiagen	8

Note

For *B. pensylvanicus*, the same specimen was divided in half and extracted using two different extraction methods.

### 2.2 DNA extraction and pre‐sequence DNA quality quantification

All specimens were extracted in Spring 2016 using Qiagen^®^ DNeasy Blood and Tissue Kit using the standard protocol with a few minor modifications to maximize DNA yield. We extracted approximately 1 cm^3^ tissue from each specimen, using thoracic tissue from *B. pensylvanicus* and *M. tepaneca*, and using the entire specimen for the smaller species *L. bardum*. We ground tissue using a MiniBeadBeater 96 (BioSpec) and 10 1.0 mm Zirconia Silica beads per sample (BioSpec 11079110z) before proceeding with lysing steps. Prior to final DNA elution we included three EtOH wash steps. We eluted DNA using Qiagen^®^ elution buffer AE warmed to 56° with two separate elution steps. We additionally investigated the DNAzol^®^ extraction technique for only *B. pensylvanicus* specimens given the larger size and ample tissue availability. *Bombus pensylvanicus *thoraces were divided in half and extracted using a customized DNAzol^®^ protocol (Chomczynski, Mackey, Drews, & Wilfinger, 1997) using the same pre‐lysis protocol as for the Qiagen extracted samples, and adding 5 µl of polyacryl carrier to 500 µl of DNAzol isolation reagent in the lysis step. Samples were washed three times with 75% EtOH and eluted in TE buffer.

We quantified DNA using both UV spectroscopy and fluorometry to measure levels of contamination and DNA concentration respectively, as recommended by Simbolo et al. (2013). Levels of potential RNA and protein contamination of the extracted DNA was measured using a NanoDrop 8000 spectrophotometer including quantification of 260/280 and 260/230 nm ratios with 1 µl of sample. A 260/280 value of 1.8 is considered “pure” DNA with little to no protein contamination, while a substantially lower value indicates the presence of protein or other contaminants that absorb light at or near 280 nm (Simbolo et al., 2013). The 260/230 ratio is a secondary measurement of DNA purity, where expected values are ~2.0–2.2 for pure DNA. A lower value indicates the presence of contaminants that absorb at 230 nm, including EDTA, carbohydrates, and phenol. Total amount of DNA per extraction was quantified using a Qubit Fluorometer (Life Technologies), with a Quant‐iT dsDNA HS assay Kit using 2 µl of sample. Qubit has been shown to be much more accurate in detecting dsDNA concentration and is less influenced by RNA contamination than spectroscopy (Simbolo et al., 2013). We additionally ran extracted DNA on a 2% agarose gel to assess levels of DNA fragmentation and weight. Individual samples were given two qualitative scores of “high” or “low” fragmentation and “high” or “low” DNA weight after visualizing the gels (Table S1, Figure S1). DNA had “high” fragmentation if there was no visible band anywhere on the gel or if visible bands were of low molecular weight (~100–200 bp). DNA had a “high” weight score if there was a visible band above 1,500 bp that was substantially darker than any band of low molecular weight (~100–200 bp). Gel qualification was only done for *B. pensylvanicus* and *M. tepaneca* as *L. bardum* samples did not visualize well enough on agarose gels to determine these qualitative measures. Each sample was diluted to equimolar concentrations, and then stored at −20°C until sequencing.

### 2.3 Library preparation and ddRAD sequencing

One hundred ng of DNA per sample after normalization using PicoGreen^®^ measurement were digested in 1X NEB Cut Smart Buffer and 100 U each EcoRI‐HF and MspI (NEB), for a final volume of 25 µl, at 37°C for 4 hr. Following a 20 min 80°C enzyme inactivation, samples were held at 12°C until ligation. We then added the following to each 25 µl digest: 3.5 µl 10X Ligase buffer (Promega), 0.5 µl T4 DNA Ligase (Promega), adapters containing 1 of 48 unique barcodes, Illumina‐compatible P5 sequences coupled to an EcoRI overhang, and Illumina‐compatible P7 sequences coupled to the MspI overhang. Plates were incubated 8 hr at 16°C and heat inactivated at 80°C for 20 min. Three pools of 45, 45 and 46 samples respectively were then made to equalize sample concentration, which was done in the order of the sample plate with each pool containing the majority of one species. These pools were mixed and combined with 0.1 volume 3 M NaAc pH 5.2 and two volumes of 100% EtOH, and then placed at −20°C for 1 hr before spinning at high speed for 10 min in a bench top microfuge. Pellets were washed twice in one mL 70% ethanol and re‐suspended in 200 µl of EB. Samples were purified with Qiagen^®^ PCR Purification columns and eluted in 2X 50 µl EB for a total of 100 µl. One volume of AMPure XP beads was added to the elutant, and then DNA was purified as per the manufacturers protocol, eluted in 35 µl EB. Thirty µl of each pool containing between 1.9 and 2.2 µg DNA was subjected to Pippin Prep size selection on a 2% agarose gel with internal size markers aiming for 270–330 base pair inserts. Recovered samples were cleaned with 1X AMPure XP beads and quantified on a DeNovix spectrophotometer. One hundred and fifty ng of each pool was then subjected to a pre‐selection PCR (PreCR) where a biotinylated forward primer and unique indexed reverse primers were used to amplify and tag desired DNA fragments. Reactions (200 µl total) contained 200 nM dNTPs, biotinylated forward and two P7‐index primers per pool, and 4 units Phusion Hi‐Fidelity Taq (NEB). Reactions were then split into 2 X 100 µl volumes for thermocycling. Following an initial denaturation at 98°C for 30 s, samples were subjected to 18 cycles of 98°C for 10 s, 58°C for 30 s, and 72°C for 30 s, with a final elongation step for 5 min at 72°C and then held at 4°C. PCR products were cleaned up in Qiagen^®^ PCR purification columns and 1X AMPure XP beads, and quantified as before. Removal of EcoRI‐EcoRI and MspI‐MspI fragments was achieved using Dynabeads/M270 Streptavidin coupled magnetic beads (ThermoFisher). Briefly, 50 µl of beads per sample were captured and washed twice with 1X Bead Washing Buffer (1X BWB, 10 mM Tris‐HCl (pH 7.5), 1 mM EDTA, 2 M NaCl). Beads were resuspended in 100 µl 2X BWB and mixed with 2,000 ng of PreCR product in 100 µl EB, and then incubated at room temperature for 20 min. Beads were then washed three times in 200 µl 1X BWB, twice in 200 µl water, and once in 100 µl 1X SSC. Beads were then resuspended in 50 µl 1X SSC and heated at 98°C for 5 min, placed on a magnet and supernatant removed. This elution was repeated and the final supernatants were cleaned up with Qiagen^®^ PCR columns. The eluted single‐stranded DNA was DeNovix quantified and diluted to 1 ng/µl with EB. A final PCR was performed on 10 ng of input ssDNA using P5 and P7 primers in a 75 µl reaction as described above but with only 8 cycles, to convert to double‐stranded DNA. Final PCR products were purified with 1X AMPure XP beads, quantified and assessed for quality on a Fragment Analyzer (Advanced Analytics). Sequencing of all 136 samples was done across two lanes of Illumina HiSeq 2500 operated by the Genomics and Bioinformatics service at Texas A&M University (TAMU), with 125 million reads per lane. De‐multiplexing of sequencing reads, trimming of adapters, and removal of barcodes, was performed using bclfastq2 v2.19 (Illumina).

### 2.4 Statistical analysis

Raw sequence data were assembled into putative loci and called to SNP's using the STACKS pipeline (v. 2.0 beta 9). To determine the optimal parameter settings for the pipeline, we used the strategy detailed by Paris, Stevens, & Catchen (2017). Briefly, we repeated the assembly across a grid of values of the “*m*”, “*M*” and “*n*” parameters (the minimum depth allowed for a putative allele, the number of mismatches allowed when merging putative alleles into putative loci, and the number of mismatches allowed when matching putative loci across samples respectively). We selected those values that resulted in the greatest number of polymorphic loci shared across 80% of the samples in a given species. These were *m* = 7, *M* = 1, and *n* = 1 for *B. pensylvanicus*, *m* = 3, *M* = 1, and *n* = 1 in *M. tepaneca*, and *m* = 3, *M* = 3, and *n* = 3 in *L. bardum*. Although the levels of depth required for accurate population genetic analysis is generally around 20× coverage (Willing, Dreyer, & Oosterhout, 2012), setting the *‐m* and *‐M* levels too high initially can cause allelic dropout when assembling ddRAD genotypes (Mastretta‐Yanes et al., 2015).

All further analyses were performed using R version 3.4.3. Because DNA quality often varied between specimens even within a treatment, we first assessed the overall success of various treatments separate from DNA quality by considering the degree of sequence similarity between samples in each treatment. We measured this by finding all possible subsets of samples of a given size, and counting the number of shared polymorphic loci within each subset. We calculated sequence similarity at three subset sizes increasing in conservatism: one sample (i.e. all samples within a treatment were compared), three samples, and six samples; thereby allowing us to assess consistency of similarity patterns between treatments. For example, a treatment with eight samples has 56 unique combinations of three samples and 28 unique combinations of six samples. Each triplet or sextet will have some number of polymorphic loci that occur in all three or six members. To determine whether sequence similarity varied substantially across treatments, we used a Kruskal‐Wallace test statistic, where each singleton/triplet/sextet is treated as an observation. However, we could not apply the usual Kruskal‐Wallace test procedure because triplets and sextets are not independent (the same pair of bees could occur in multiple triplets/sextets). Instead, we simulated the distribution of the test statistic under the null hypothesis that individual samples are exchangeable across treatments. We repeatedly permuted samples across treatments, re‐computing the value of the test statistic in each permutation, and computed an approximate *p*‐value as the proportion of simulated test statistics that are greater in magnitude than our observed test statistic. To determine how individual treatments differed, we repeated the simulation procedure on a pairwise basis using Dunn's tests and corrected for multiple comparisons using Bonferonni‐Holm correction (Holm, 1979).

Second, to assess how treatment and DNA quality jointly influenced sequencing success within samples, we considered two metrics of assembly quality that are measured for each locus/sample combination: (a) the successful recovery of a particular locus within a particular sample (a binary variable: locus was present/absent in a sample); (b) the sequencing depth of a successfully recovered locus in a sample (a non‐zero count). We used generalized linear mixed models (GLMMs) to estimate the expected probability of locus recovery and the average sequencing depth across treatments, and as a continuous function of DNA quality. We modeled locus recovery as a binomial random variable of size one (with a logit link) and locus sequencing depth as a zero‐truncated negative binomial random variable (with a log link). We additionally modeled locus sequencing depth per million reads by including an offset (e.g. log millions of reads per sample) in the negative binomial model to standardize effects across samples and treatments with different numbers of reads. All models included a random intercept per locus to capture variability in sequencing success across the genome, and a random intercept per individual specimen in *B. pensylvanicus* (the same specimens were subjected to both extraction methods in this species). Preliminary analyses on *B. pensylvanicus* indicated that extraction method distinctly affected ddRAD results from different treatments, and so an interaction between extraction method and treatment was also included in models for this species. Because of the inherent repetition in our *B. pensylvanicus* dataset, we were able to count the number of loci that were recovered in both extractions from the same specimen (out of the total number of loci recovered for that specimen) and test for differences in rates of repeated locus recovery between treatments, where we modeled this count with a binomial generalized linear model (e.g. Mastretta‐Yanes et al., 2015). As qualitative levels of fragmentation per sample were unobtainable for many specimens and preliminary analyses indicated that these were not significant in any of the exploratory models, this metric was removed from further analysis. In practice, RADseq assemblies are filtered to remove loci that occur in less than a given fraction of specimens (often 60% or 80%, e.g. Maroso et al., 2018), as these loci could be spurious or originate from exogenous material. To assess the sensitivity of our results to various filters, we refit the models to subsets of the data resulting from removing loci that occurred in less than 40%, 60% and 80% of specimens per species. We assessed the explanatory power of overall treatment/quality effects using ANOVA, and conducted post‐hoc comparisons between individual treatments using the simultaneous testing procedure implemented in the R package multcomp (Hothorn, Bretz, & Westfall, 2008). Any significant differences between treatments for DNA quality metrics were tested by ANOVA and post‐hoc Tukey tests correcting for multiple comparisons.

## 3 RESULTS

After quality filtering and assembly steps were completed in STACKS, all species, treatments, and extraction methods showed a mean number of highly reliable loci that would be adequate for informative population genetics analyses (minimum of ~1,000 loci, as suggested by Willing et al., 2012). *Bombus pensylvanicus *specimens showed a mean (±*SE*) of 4,415.0 (±239.0) polymorphic loci with a mean locus depth of 296.8 (±23.2) across all treatments and extraction methods. *Melissodes tepaneca *specimens showed a mean (±*SE*) of 11,528.0 (±177.3) polymorphic loci with mean locus depth (±*SE*) of 66.9 (±4.2) across all sample/storage treatments, and *L. bardum* specimens showed a mean (±*SE*) of 16,424.3 (±733.3) polymorphic loci with a mean locus depth of 42.5 (±4.3) across all sample/storage treatments. Mean loci and depth, as well as DNA quality metrics, also varied between treatments within species (Figure 1, Table 2).

**Table 2:** Mean (±*SE*) DNA quality and ddRAD assembly quality metrics per species per treatment

Species	Treatment	Nanodrop 260/280	Nanodrop 260/230	DNA concentration (ng/μl)	Mean polymorphic loci	Mean locus depth
*Bombus pensylvanicus*	Net‐EtOH	2.1 ± 0.02	1.7 ± 0.1	10.9 ± 0.1	5,467.6 ± 772.5	453.6 ± 64.3
	Net‐Dry	1.9 ± 0.1	1.5 ± 0.2	12.2 ± 0.4	3,748.3 ± 205.8	175.0 ± 37.0
	Vane‐Dry	2.0 ± 0.04	2.1 ± 0.04	13.0 ± 0.4	3,830.1 ± 79.3	286.2 ± 20.6
*Melissodes tepaneca*	Net‐EtOH	2.0 ± 0.02	1.9 ± 0.1	14.4 ± 0.8	11,646.4 ± 202.4	104.7 ± 4.8
	Net‐Dry	2.1 ± 0.1	1.7 ± 0.1	13.1 ± 0.7	11,876.3 ± 383.9	45.3 ± 3.5
	Vane‐Gly	2.0 ± 0.02	2.1 ± 0.1	12.2 ± 0.5	10,370.0 ± 299.8	87.8 ± 2.6
	Vane‐Dry	2.0 ± 0.02	1.9 ± 0.1	12.7 ± 0.3	12,126.6 ± 471.9	50.9 ± 3.4
	Pan	2.0 ± 0.02	2.0 ± 0.1	12.4 ± 0.5	11,476.0 ± 338.9	48.2 ± 3.3
*Lasioglossum bardum*	Net‐Dry	1.9 ± 0.04	1.0 ± 0.1	10.5 ± 1.0	13,468.4 ± 813.5	18.5 ± 1.1
	Vane‐Gly	2.1 ± 0.01	1.7 ± 0.1	13.4 ± 1.2	12,161.4 ± 191.1	72.7 ± 10.1
	Vane‐Dry	1.7 ± 0.2	1.2 ± 0.1	10.1 ± 1.1	19,385.2 ± 1,246.9	34.8 ± 4.1
	Pan	2.2 ± 0.01	2.0 ± 0.02	12.8 ± 0.3	20,311.3 ± 560.9	49.0 ± 4.1

Note

All specimens were extracted using Qiagen DNeasy kits. For results for DNAzol extracted bees, see Table S2.

Mean loci did not differ notably between extraction methods across treatments (*B. pensylvanicus* only): DNAzol extracted specimens had 4,339.9 (±323.1) mean loci, with 267.8 (±53.5) mean depth and Qiagen extracted specimens had 4,490.4 (±357.2) mean loci with 304.9 (±81.0) mean depth. Because extraction method did not have a prominent effect on overall numbers of loci, patterns of sequence similarity, retained loci, or depth, we henceforth only report results of Qiagen extracted specimens in the main text. See Tables S2‐S3 and Figures S2‐S4 for results including DNAzol extracted *B. pensylvanicus* specimens. Additionally, treatment did not significantly predict repeated locus recovery across multiple extractions of the same *B. pensylvanicus* specimen (Figure S4).

### 3.1 Sequence similarity

We assessed the amount of sequence similarity (number of shared loci within a given subset of samples) between specimens within each treatment and species, allowing us to understand the overall effect of each treatment on ddRAD assembly quality, separate from any DNA quality effects. Here we report only the results of the intermediate‐level conservative filter (three samples per treatment), as Kruskal‐Wallace tests indicated similar patterns of sequence similarity between treatments within a species regardless of sample filter. See Supporting Information and Figure S2 for results for the one‐ and six‐sample filters.

There was no significant difference in levels of sequence similarity between treatments in *B. pensylvanicus* (*H* = 87.63, *p* = .270). Treatment had a marginally significant effect on sequence similarity for *M. tepaneca* (*H* = 128.172, *p* = .102), but post‐hoc Dunn's tests did not show any significant differences between treatments after Holm correction for multiple comparisons. Treatment had a significant effect on sequence similarity in *L. bardum* (*H* = 239.33, *p* < .001), with specific differences between treatments highlighted below.

#### 3.1.1 Net‐EtOH

**Figure 2:**
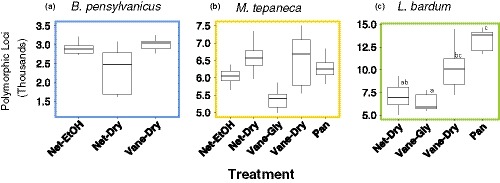
Box plots showing numbers of polymorphic loci retained between specimens (sequence similarity) when grouping by three random samples per species. Different letters indicate statistically significant differences (Dunn's, *p* < .05) between treatments in *Lasioglossum bardum*. There were no statistically significant differences in sequence similarity between treatments for either *Bombus pensylvanicus* or *Melissodes tepaneca* after correcting for multiple comparisons. Sample treatments are coded as in Table 1, and graphs are color‐coded according to species as in Figure 1

Net‐EtOH specimens showed intermediate levels of sequence similarity as compared to the other treatments in *B. pensylvanicus* and *M. tepaneca* (Figure 2a,b), but sequence similarity was not significantly different in Net‐EtOH from the other treatments in either species.

#### 3.1.2 Net‐Dry

Levels of sequence similarity within specimens were quite variable between species. Net‐Dry had the lowest level of similarity in *B. pensylvanicus* (Figure 2a), the highest level of similarity in *M. tepaneca* (Figure 2b), and the second‐lowest level of similarity for *L. bardum* (Figure 2c). Net‐Dry specimens had significantly lower sequence similarity than Pan in *L. bardum* (*p* = .008), but was not significantly different from any other treatment.

#### 3.1.3 Vane‐Gly

Vane‐Gly specimens had the lowest level of sequence similarity for both *M. tepaneca* and *L. bardum* (Figure 2b,c). In *L. bardum*, Vane‐Gly specimens showed significantly lower sequence similarity than both Pan (*p* < .001), and Vane‐Dry (*p* < .001).

#### 3.1.4 Vane‐Dry

In *B. pensylvanicus*, these specimens showed the highest level of sequence similarity (Figure 2a), although sequence similarity in Vane‐Dry was not significantly different from the other two treatments. Vane‐Dry specimens showed intermediate levels of sequence similarity in both *M. tepaneca* and *L. bardum* (Figure 2b,c). In *L. bardum*, Vane‐Dry specimens had significantly greater levels of similarity than Vane‐Gly (*p* < .001), but not in the other two treatments.

#### 3.1.5 Pan

Pan‐trapped specimens showed the second‐highest level of sequence similarity for *M. tepaneca* (Figure 2b), although post‐hoc tests did not reveal a significant difference in treatments for the three‐sample filter. Pan showed the highest level of sequence similarity in *L. bardum* (Figure 2c), and was significantly different from Vane‐Gly (*p* < .001) and Net‐Dry (*p* = .008).

### 3.2 Treatment and DNA quality effects on locus recovery and depth

When considering how treatment affected ddRAD assembly quality in conjunction with pre‐sequence DNA quality, GLMM analyses revealed that levels of locus recovery (probability that a locus occurs in other samples), depth per locus (average number of reads in which a particular locus was found), and standardized depth (average locus depth per million reads) varied between treatments and species. Patterns of locus recovery and both depth measurements were similar within species across sample filters, which included one sample (i.e. all loci across samples), and shared among a minimum of 40%, 60%, and 80% of samples respectively. Standardizing average depth by millions of raw reads indicated that the differences in average depth among treatments generally tracked differences in numbers of raw reads (Figures S3 and S5). Therefore, we consider raw read yield an important part of an overall treatment effect and thus primarily report the results of the locus recovery and average depth per locus models in the main text, reporting the 60% sample filter only. See Supporting Information for reporting of standardized depth models, Tables S3‐S5 for full results of all models, and Figures S3 and S5 showing recovered loci, locus depth, and standardized depth for each species and treatment across all sample filters.

#### 3.2.1 Net‐EtOH

**Figure 3:**
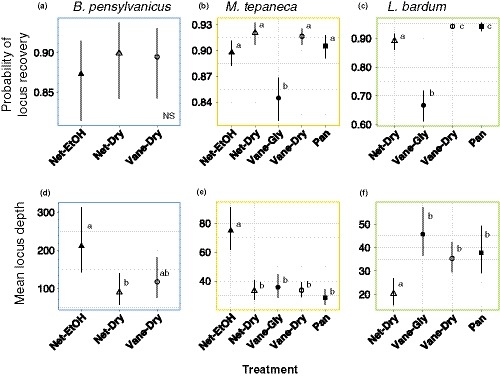
Treatment effects on ddRAD assembly quality for Qiagen extracted *Bombus pensylvanicus* (a, d), *Melissodes tepaneca* (b, e) and *Lasioglossum bardum* (c, f), filtered by a minimum sample of 60% of specimens per species. (a–c) Number of shared loci, measured as scaled probability of a locus occurring in another random sample. (d–f), Average depth per locus. Bars represent 95% confidence intervals. Different letters indicate statistically significant differences (Tukey HSD, *p* < .05) between treatments. Treatments are coded as in Table 1, and represented by different markers: Net‐EtOH: filled triangles, Net‐Dry: unfilled triangles, Vane‐Dry: unfilled circles, Vane‐Gly: solid circles, and Pan: solid squares. Graphs are color coded according to species as in Figure 1

Net‐EtOH specimens did not have significantly different locus recovery from the other two treatments in *B. pensylvanicus* (Figure 3a). Net‐EtOH specimens had higher locus recovery than Vane‐Gly *M. tepaneca* specimens (*z* = 3.587, *p* = .002, Figure 3b), but was not significantly different from the other four treatments in this species.

However, Net‐EtOH specimens showed differences in depth of coverage from other sampling treatments in both *B. pensylvanicus* and *M. tepaneca*. Locus depth was significantly higher than the Net‐Dry treatment in *B. pensylvanicus* (*z* = 2.847, *p* = .033, Figure 3d), and had significantly higher depth than all other treatments in *M. tepaneca*, (*z* = 4.660, *p* < .001, Figure 3e).

#### 3.2.2 Net‐Dry

Net‐Dry specimens did not differ significantly from the other two treatments in locus recovery for *B. pensylvanicus* (Figure 3a). However, Net‐Dry specimens showed significantly higher locus recovery than Vane‐Gly specimens for both *M. tepaneca* (*z* = 5.362, *p* < .001, Figure 3b) and *L. bardum* (*z* = 7.073, *p* < .001, Figure 3c). However, despite somewhat higher levels of locus recovery, Net‐Dry specimens tended to have lower levels of depth across all three species. Net‐Dry specimens had significantly lower levels of depth than Net‐EtOH for both *B. pensylvanicus* (as noted above) and *M. tepaneca* (*z *= −5.865, *p* < .001), but otherwise did not differ significantly from the other treatments in these species (Figure 3a,b). Net‐Dry specimens also had the lowest level of depth for *L. bardum* (Figure 3e), significantly lower than all other treatments in this species, including Pan (*z* = −2.654, *p* = .037), Vane‐Dry (*z* = −3.368, *p* = .004) and Vane‐Gly (*z* = −4.163, *p* < .001).

#### 3.2.3 Vane‐Gly

Vane‐Gly specimens had variable results between levels of locus recovery and depth of coverage, as well as between species. Vane‐Gly specimens had the poorest locus recovery, significantly lower than all other treatments for both species in which the method was used (*M. tepaneca* and *L. bardum* [Figure 3b,c; Tables S4 and S5]). For *M. tepaneca*, Vane‐Gly specimens also had significantly lower depth than Net‐EtOH specimens (*z* = −4.660, *p* < .001), but otherwise did not differ significantly from the other three treatments (Figure 3e). For *L. bardum*, however, Vane‐Gly had the highest level of depth compared to the other three treatments but was only significantly higher in depth than Net‐Dry specimens (*z* = 4.163, *p* < .001, Figure 3f).

#### 3.2.4 Vane‐Dry

Vane‐Dry specimens did not differ significantly in locus recovery or depth from the other two treatments used in *B. pensylvanicus* (Figure 3a,d). However, Vane‐Dry specimens had the second‐highest and highest levels of locus recovery for both *M. tepaneca* and *L. bardum* respectively. Vane‐Dry specimens had significantly higher locus recovery than Vane‐Gly (*z* = 6.351, *p* < .001) for *M. tepaneca*, and significantly higher locus recovery than Net‐Dry (*z* = 4.622, *p* < .001), as well as Vane‐Gly for *L. bardum* (*z* = 17.897, *p* < .001, Figure 3c). Vane‐Dry had significantly lower levels of depth than Net‐EtOH for *M. tepaneca* (*z* = 6.649, *p* < .001), but was not significantly different in depth from the other three treatments (Figure 3e). Vane‐Dry had significantly higher levels of depth than Net‐Dry (*z* = 4.163, *p* < .001) in *L. bardum*, and did not differ from the other two treatments in depth (Figure 3f).

#### 3.2.5 Pan

Pan‐trapped specimens had significantly higher locus recovery than Vane‐Gly for *M. tepaneca* (*z* = 4.861, *p* < .001, Figure 3b), and significantly higher locus recovery than Vane‐Gly (13.283, *p* < .001) and Net‐Dry (7.073, *p* < .001) for *L. bardum* (Figure 3c). Pan specimens did not differ significantly in locus recovery from the best treatment for either species. Pan specimens had the lowest level of average depth for *M. tepaneca* but did not differ significantly from any treatment other than Net‐EtOH for *M. tepaneca* (*z* = −6.787, *p* < .001, Figure 3e). For *L. bardum*, Pan specimens showed significantly higher average depth than Net‐Dry specimens (*z* = 2.654, *p* = .037) and did not differ significantly from the other treatments (Figure 3f).

#### 3.2.6 DNA concentration

Higher sample DNA concentration was significantly associated with higher locus recovery and locus depth in *B. pensylvanicus* (loci: *z* = 2.258, *p* = .024, depth: *z* = 2.820, *p* = .005), and higher locus recovery in *L. bardum* (*z* = 4.370, *p* < .001). Surprisingly, DNA concentration had a significant *negative* effect on *M. tepaneca* sample depth (*z *= −2.350, *p* = .019), likely driven by a few outlier samples which had high DNA concentration but low locus depth. DNA concentration was not significantly affected by treatment in *B. pensylvanicus* or *M. tepaneca*. DNA concentration was marginally significantly related to treatment in *L. bardum* (*F* = 2.798, *df* = 3, *p* = .056), with Vane‐Dry having marginally significantly higher DNA concentration than Vane‐Gly (*p* = .097).

#### 3.2.7 Nanodrop indices

*Bombus pensylvanicus* samples with higher 260/280 indices had significantly higher locus recovery (*z* = 3.180, *p* < .001) and higher depth (*z* = 2.490, *p* < .013). 260/280 ratios were significantly related to treatment in *B. pensylvanicus* (*F* = 6.493, *p* = .005), with Net‐Dry samples having significantly lower 260/280 ratios than Net‐EtOH samples (*p* = .004) and marginally significantly lower ratios than Vane‐Dry samples (*p* = .066). There was no significant effect of either NanoDrop 260/280 or NanoDrop 260/230 on number of shared loci or depth for *M. tepaneca*. For *L. bardum*, the other NanoDrop index 260/230 was significantly positively related to higher locus recovery (*z* = 2.03, *p* = .042). 260/230 was also significantly related to treatment in *L. bardum* (*F* = 25.8, *df* = 3, *p* < .001), with post‐hoc Tukey tests revealing several differences between treatments. Specifically, both Pan and Vane‐Gly specimens had significantly larger 260/230 ratios than Vane‐Dry specimens (*p* < .001) and Net‐Dry specimens (*p* < .001).

## 4 DISCUSSION

Our results reveal very few differences between the ddRAD sequencing success of bee samples collected across a variety of traditional entomological collection methods; this bolsters previous work showing that field‐collected and traditionally curated samples of multiple non‐model species can be utilized for population genetic analyses using the ddRAD protocol (Tin et al., 2014; Vaudo et al., 2018). While we documented lower DNA concentrations and higher contamination levels in many treatments as compared to typical fresh DNA extractions, this did not prohibit retrieval of thousands of polymorphic loci and high levels of locus depth. However, we show that storage and sampling methods can have significant effects on these metrics of ddRAD assembly success, where treatment effects were distinct between the DNA quality and locus recovery/depth metrics and also between species. Additionally, our analyses indicate that extraction method has only a small effect on ddRAD assembly quality, with only minor differences in overall loci and depth. Lastly, we show that DNA concentration alone is not always predictive of higher quality ddRAD assemblies, and that other measures of DNA quality such as Nanodrop indices may be particularly useful for predicting the likelihood of downstream success in ddRAD projects.

To begin with, in all treatments and across species, enough loci (≿4,000) with sufficient coverage (≿20×) were obtained to conduct highly informative population genetic studies (Andrews & Luikart, 2014; Willing et al., 2012). Specifically, we found that, between many collection and storage treatments, there was often no significant effect on numbers of loci and depth, which was also consistent between species of differing body size. This was especially encouraging for our smallest species tested (*L. bardum*) as the amount of genetic material extracted was expected to be quite low and thus potentially more sensitive to DNA degradation. Interestingly, a study investigating genotyping success on museum curated spiders found that genotyping quality actually declined with increasing body size. Krehenwinkel and Pekar (2015) suggest that smaller specimens are likely more quickly preserved than larger specimens, which may lead to good DNA recovery. Our results may also reflect this finding, and are congruent with other studies that have shown successful NGS success of museum specimens of small insects (Sproul & Maddison, 2017), as well as those utilizing ddRAD to successfully sequence traditionally collected and curated bees (Vaudo et al., 2018). Vaudo et al. (2018) specifically found that specimen age did not affect number of polymorphic loci or coverage depth, and similarly showed that collection method had the most significant effects on overall ddRAD assembly quality.

While we acknowledge that the treatments in our study utilized different time and handling procedures, they represented standard techniques in insect collecting and curation (LeBuhn et al., 2003; Stephen & Rao, 2007), and we expected their utility for ddRAD to vary considerably. Specifically, we predicted that specimens that were netted directly into and stored in EtOH (Net‐EtOH) would show the best results in terms of higher numbers of loci, greater average locus depth, and lower levels of sequence similarity. Indeed, Net‐EtOH specimens showed significantly greater average depth than other treatments in the two species where this treatment was used (*B. pensylvanicus* and *M. tepaneca*), but was surprisingly similar to other treatments in terms of number of loci, sequence similarity, and standardized depth. In contrast, specimens that were netted using ethyl acetate kill jars and then frozen at −20°C before pinning (Net‐Dry), often showed lower quality assemblies, especially in quantification of average locus depth. Although we predicted that the −20°C freezing step would preserve DNA well, it is important to note that these specimens were held on ice in the field for up to 8 hr prior to freezing. Thus, it is likely that these Net‐Dry specimens did not have the opportunity to dry quickly enough to delay DNA degradation before freezing. While some studies suggest that ethyl acetate contributes to degraded DNA (Dillon et al., 1996), others have suggested that such degradation is likely due to samples remaining in a prolonged damp state after ethyl acetate exposure before being pinned (Quicke, Belshaw, & Lopez‐Vaamonde, 1999). Quick desiccation of entomological specimens has been shown to preserve DNA similarly well to preservation in EtOH (Quicke et al., 1999), and it is likely that we are documenting related patterns in our ddRAD assemblies. The exception to this general pattern in our results was that *L. bardum* Net‐Dry specimens showed low average locus depth (overall low numbers of reads), but high standardized depth (average depth per locus per million reads) than other treatments. Again, this result is likely due to the faster desiccation rates of smaller specimens better preserving DNA (as discussed above). Thus, Net‐Dry *L. bardum* specimens had more reliable locus recovery per standard number of reads than the other larger species, even if overall locus depth was low in this treatment.

Surprisingly, in contrast to our predictions, we found that specimens collected into propylene glycol traps (Vane‐Gly) produced lower quality ddRAD assemblies, and that both blue vane trapped specimens with no preservative (Vane‐Dry) and pan‐trapped specimens into soapy water (Pan) produced relatively high quality ddRAD assemblies. Our results were unexpected because past studies report glycol as an effective DNA preservative for marker‐based studies (Dillon et al., 1996; Ferro & Park, 2013; Rubink et al., 2003). It is possible that differences in glycol concentration (some studies utilized lower concentrations of glycol) or glycol type (Dillon et al. (1996) utilized ethylene rather than propylene glycol) contributed to the disparity between our results and past work. However, we believe this is unlikely given that both Ferro and Park (2013) and Rubink et al. (2003) showed no differences between DNA quality and sequencing success between specimens preserved in differing concentrations of propylene glycol. Overall, these results suggest that propylene glycol preserves DNA effectively for marker‐based studies, but not as well for ddRAD sequencing. Mechanistically, it is also possible that slower desiccation affected the propylene glycol samples, because residual glycol could have prevented EtOH from fully penetrating the specimen in the intermediate preservation step, thus inhibiting quick drying in subsequent pinning. This mechanism is supported by the fact that we documented higher average locus depth and standardized depth in Vane‐Gly *L. bardum,* suggesting that DNA quality may be maintained in smaller species preserved in glycol, possibly because EtOH may better penetrate these smaller specimens. We suggest that the propylene glycol method should be used with caution, with extra care taken to thoroughly wash specimens of the glycol preservative, rinsing specimens in 100% EtOH before long‐term preservation methods are conducted.

In contrast to the propylene glycol‐preserved specimens, quick desiccation may have contributed to higher‐than‐expected DNA quality in pan‐trapped and dry blue vane‐trapped specimens. Vane‐Dry often had the best results among all treatments, in some cases significantly surpassing Net‐EtOH specimens in terms of number of polymorphic loci and higher sequence similarity; this was fairly consistent across species. This was surprising as Vane‐Dry specimens were left in the trap for up to 5 days during the summer months, where ambient temperatures could be as high as 40°C. Although we originally predicted that this would cause high levels of DNA degradation, high temperatures may have allowed for quick drying of specimens leading to the preservation of higher quality DNA. Other studies have similarly shown that desiccation of specimens in silica gel can lead to high quality DNA preservation similar to freezing (Quicke et al., 1999), and that drying specimens in the sun is similar to chemical methods (Post, Flook, & Millest, 1993). Our pan‐trapped specimens, despite remaining in water for up to 24 hr, showed better‐than‐expected DNA quality and ddRAD assembly results. This is likely due to the fact that these specimens were stored in 100% EtOH immediately after the 24 hr of field time, typical of pan‐trapping protocols (e.g. Grootaert, Pollet, Dekoninck, & Achterberg, 2010); acting to rapidly dehydrate the samples. Another study has shown that pan trapped bee specimens had similar high quality ddRAD results in comparison to other capture methods, also likely due to rapid desiccation via EtOH storage (Vaudo et al., 2018).

Interestingly, we also found that DNA concentration was not always a key predictor in ddRAD sequencing success. Specifically, we found that higher DNA concentration was significantly related to higher locus depth in *B. pensylvanicus*, but not in the other two species. This was somewhat surprising as a high concentration of high‐molecular weight DNA has frequently been assumed to be necessary for high‐quality ddRAD assemblies (Andrews et al., 2016; Puritz et al., 2014). It is possible that we did not see correlations between DNA concentration and sequencing success due to exogenous DNA in our samples, however, as we analyzed the data with various sample number filters that show similar patterns, we believe that the presence of exogenous sequences is not a major driver of our results. It is also possible that the Qubit measurement was not sensitive enough to detect a correlation between DNA concentration and sequencing success, and thus we suggest that future studies include analysis with a fragment analyzer and/or other DNA quantification methods (i.e. Picogreen^®^) to assess levels of fragmentation and DNA concentration after extraction. Regardless, our results support the idea that moderate levels of DNA degradation can still produce acceptable ddRAD results (also seen in Graham et al., 2015; Tin et al., 2014). We also found that Nanodrop 260/280 and 260/230 ratios were positively correlated with greater average and standardized depth in *B. pensylvanicus* and *L. bardum* respectively. Net‐EtOH samples had highest 260/280 ratio in *B. pensylvanicus* and Pan had the highest 260/230 ratios in *L. bardum*, although the relationship between the 260/230 index and treatment was weaker in the latter species. We suggest that NanoDrop metrics should be utilized in addition to assessment of DNA concentration to assess likelihood of ddRAD assembly success for individual samples.

When comparing two DNA extraction methods, Qiagen DNeasy^®^ and DNAzol^®^, for the same specimens, we found no difference in locus recovery or average depth between the two extraction methods, although Qiagen extracted samples showed higher standardized depth. We also found some differences in sequence similarity for the Net‐Dry specimens, with Qiagen extracted specimens showing significantly more similarity than DNAzol specimens at the lower sample filters. Chen, Rangasamy, Tan, Wang, and Siegfried (2010) similarly found little difference between DNAzol and Qiagen extracted insect specimens in DNA yield. Similar to Corcoll et al. (2017), we found higher levels of salt contamination with DNAzol extracted samples showing 260/230 ratios substantially lower than 2.0 in DNAzol extracted specimens, but this did not cause major differences in ddRAD assembly quality in our study. While Qiagen kits are convenient and more time‐efficient than other methods, they are also more expensive, costing roughly twice the price of DNAzol extractions per sample (Chen et al., 2010). DNAzol extractions have also been shown to be more effective than other low‐cost extraction methods (e.g. Chelex, phenol‐chloroform) for extracting high quality DNA from museum specimens (Junqueira, Lessinger, & Espin, 2002). Therefore, we suggest that labs wishing to cut costs for DNA extraction and ddRAD sequencing can utilize the lower‐cost DNAzol protocol for DNA extractions without sacrificing ddRAD sequence quality.

Finally, we found that in some cases, there appeared to be a trade‐off between locus recovery and depth of coverage, possibly related to both sequencing bias and sample quality. Specifically, our results show that in several cases, treatments that showed high probability of locus recovery, showed lower levels of depth and vice versa. For example, Net‐Dry specimens for both *M. tepaneca* and *L. bardum* showed higher probabilities of locus recovery, but lower levels of average depth as compared to other sampling and storage treatments. This pattern may reflect what was detected by DaCosta and Sorenson (2014), who found that fragment length and depth had a negative relationship in ddRAD, where sequencing depth peaked at 200–225 base pair fragment length, and declined at 325 base pairs. This implies that greater fragmentation in template DNA could lead to higher depth for the smaller fragments, due to more overall molecules contained in the smaller fragment length distribution, but fewer overall loci amplified across different fragment sizes. Thus, our study supports this tradeoff, and we suggest that researchers be sure to utilize sampling treatments that balance high levels of both number of loci and sequencing depth. Some researchers suggest that depth is one of the most important factors to consider in NGS analyses, and that labs should not sacrifice depth for more loci or number of samples (Andrews & Luikart, 2014). This is because high levels of coverage allow for the reduction or elimination of missing data, which is more common in highly degraded samples (Mikheyev et al., 2017).

In conclusion, by testing traditional collection and storage protocols commonly used by entomologists, we show that many of these techniques can yield specimens suitable for ddRAD sequencing and analysis. Based on our results, we conclude that although netting directly into 100% EtOH produces high quality ddRAD assemblies, trapping methods that allow for quick desiccation and subsequent preservation of specimens can also be utilized for successful ddRAD sequencing. This allows researchers greater flexibility in utilizing trapping methods which may be more convenient if, for example, encounter rates are low for the species of interest, or if specimens are not feasibly captured via netting. Passive trapping may also be an advantage in some studies, because hand netting is often biased towards larger bodied insects, and is also dependent on the researcher's experience and skill (Roulston et al., 2007). We also show that pinned specimens can be used to build successful ddRAD assemblies, and thus we posit that museum collections hold great opportunity for ddRAD‐based research, especially considering the advent of new low‐invasive sampling methods where the physical specimen can be retained (Andersen & Mills, 2012; Thomsen et al., 2009; Vaudo et al., 2018). For example, ddRAD could be used to examine population genomic patterns in insect specimens collected over long periods of time, a process which may be key to better understanding global pollinator declines (Cameron et al., 2011). We suggest that specimens netted directly into EtOH, as well as pan trapped and blue vane trapped specimens without preservatives, should be first‐choice specimens for conducting NGS projects. Once DNA is extracted, concentration as well as DNA purity measurements could be used to screen samples for likelihood of downstream assembly success. Overall, our results encourage the expansion of genetic monitoring via NGS to a wider variety of non‐model species in order to address new and exciting research questions in species and specimens previously believed to be too rare and degraded to investigate.

## CONFLICT OF INTEREST

None declared.

## AUTHOR CONTRIBUTIONS

K.M.B., A.R.C., S.C., and S.J. conceived and designed experiments. K.M.B. and S.C. collected and preserved specimens; K.M.B, A.R.C., S.C., and R.M. performed experiments. K.M.B. and N.S.P. analyzed the data. S.J. contributed materials and analysis tools. K.M.B. and S.J. wrote the manuscript.

## Data Availability

Sequence assembly files (.vcf and output from STACKS), code to fit models, and a .csv file of treatments, sample ID's, sequence ID's and DNA quality metrics is deposited on DRYAD, https://doi.org/10.7291/D1CD4P. Raw sequence data is deposited on GenBank, accession numbers SAMN12836825‐SAMN12836960.
