# Supplementary material for: Utilizing field collected insects for next generation sequencing: Effects of sampling, storage, and DNA extraction methods
Source: Ecol Evol. 2019 Dec 3;9(24):13690–705. doi: 10.1002/ece3.5756 (PMC6953651; doi:10.1002/ece3.5756)
Supplement: Supplementary file 1 [file ECE3-9-13690-s001.pdf]

## Supplemental Information for:

### Utilizing field collected insects for next generation sequencing: effects of sampling, storage, and DNA extraction methods

**Kimberly M. Ballare[1,2,7], Nathaniel S. Pope[1,3], Antonio R. Castilla[1,4], Sarah Cusser[1,5], Richard P. Metz[6], Shalene Jha[1]**

[1] Department of Integrative Biology, The University of Texas at Austin, 205 W 24th Street, 401 Biological Laboratories, Austin, TX 78712, USA
[2] Current Affiliation: Department of Ecology and Evolutionary Biology, University of California Santa Cruz, Santa Cruz, CA 95060, USA
[3]Current Affiliation: Department of Entomology, Pennsylvania State University, University Park, PA 16802, USA
[4] Current Affiliation: Centre for Applied Ecology "Prof. Baeta Neves"/INBIO, Instituto, Superior of Agronomy, University of Lisbon. Tapada da Ajuda, 1349-017, Lisbon, Portugal.
[5] Current Affiliation: Kellogg Biological Station, Michigan State University, 3700 East Gull Rd., Hickory Corners, MI 49060, USA
[6] Genomics and Bioinformatics Service, Texas A&M AgriLife Research, College Station, TX 77845, USA
[7] Author for correspondence

## Table of Contents


| | |
|---|---|
| **Results for sequence similarity for one- and six-sample filters (Qiagen extracted)** | Page 2 |
| **Results for standardized depth m,odels** | Page 2 |
| **Results for DNAzol extracted *B. pensylvanicus* specimens** | Page 3 |
| **Table S1** | Page 4 |
| **Table S2** | Page 6 |
| **Table S3** | Page 7 |
| **Table S4** | Page 10 |
| **Table S5** | Page 13 |
| **Fig. S1** | Page 15 |
| **Fig. S2** | Page 16 |
| **Fig. S3** | Page 17 |
| **Fig. S4** | Page 18 |
| **Fig. S5** | Page 19 |

**Sequence similarity for one- and six-sample filters (Qiagen extracted)**

*B. pensylvanicus*

There was no significant difference in levels of sequence similarity between treatments of *B. pensylvanicus* in any binning group, although there was a marginally significant effect of treatment on sequence similarity when comparing all samples (binned by one sample, H=5.13, p=0.075). Post-hoc Dunn's tests with Holm correction for multiple comparisons revealed that Net-EtOH had significantly higher levels of sequence similarity than Net-Dry specimens in the one-sample bin (p=0.048), but no other differences between treatments were significant. In general, Vane-Dry showed the highest level of similarity for all sample filters, including the most conservative filter of six samples per treatment (Fig. S2b).

*M. tepaneca*

Treatment had a significant effect on sequence similarity when binned by one sample (H=12.779, p=0.010), but not when binned by six samples (H=88.14, p=0.241). Post-hoc Dunn's tests did not show any significant differences between treatments in *M. tepaneca* after Holm correction for multiple comparisons. Vane-Gly treatment showed the lowest numbers of similar loci than all other treatments, with Net-Dry, Pan, and Vane-Dry showing the highest levels of similarity in *M. tepaneca* for all sample filters including the most conservative filter (Fig. S2c).

*L. bardum*

There was a significant effect of treatment on sequence similarity when binned by one sample (H=21.63, p<0.001) and six samples (H=256.91, p=0.005). Post-hoc Dunn's tests with Holm correction for multiple comparisons indicate that Pan specimens had significantly more similar loci than both Net-Dry and Vane-Gly in the one-sample filter (p < 0.001), but not in the six-sample filter (Pan vs Net-Dry: p=0.276, vs Vane-Gly: p=0.350). Dunn's tests also showed that Vane-Dry specimens had significantly more sequence similarity than Vane-Gly and Net-Dry specimens in the one-sample filter (p<0.001), but not in the six-sample filter (p=0.350). In general, Pan specimens showed the highest level of sequence similarity among all treatments and in all sample filters (Fig. S2d).

**Standardized Depth Models**

As in the main text, here we report results for the 60% sample filter. See Tables S3-S5 for results of all models and sample filters. For the most part, we found that standardizing depth by millions of raw reads evened out differences among treatments seen in models of average (overall) locus depth. Indeed, there were no significant effects of treatment on standardized depth in either *B. pensylvanicus* (Fig S3e, f) or *M. tepaneca* (Fig S5e) after correcting for multiple comparisons. However, for *L. bardum* we did see that Net-Dry and Vane-Gly specimens had significantly higher standardized depth than both Vane-Dry (Tukey HSD, p <0.001) and Pan (Tukey HSD, p=0.004 and p <0.001 respectively, see Fig S5f).

## DNAzol extracted *B. pensylvanicus* specimens

*Sequence similarity*

Kruskal-Wallace tests indicated that treatment had an effect on sequence similarity in the DNAzol extracted *B. pensylvanicus* specimens when comparing all samples (binned by one sample, H=9.765, p=0.009) and binned by three samples (H=189.87, p=0.029), unlike the Qiagen extracted samples which did not show a significant difference in sequence similarity between treatments. Dunn's tests revealed that Net-Dry specimens had significantly less recovered loci than Net-EtOH specimens in the one-sample filter (p=0.006). Additionally, when comparing different extraction methods within treatments, there were significant differences between the Qiagen extracted and the DNAzol extracted Net-Dry specimens at the one- and three-sample filters, where Qiagen extracted Net-Dry specimens showed slightly but significantly more matching loci than the DNAzol extracted Net-Dry specimens (Sign test, 1 sample: p=0.018; 3 samples: p=0.022). There were no other significant differences between extractions for the other two treatments. Notably, Qiagen extracted Net-EtOH specimens were more variable in the levels of sequence similarity than DNAzol extracted Net-EtOH specimens at the six-sample filter (Fig. S2).

*Loci and Depth*

As in the main text, here we only report the results of the 60% sample filter. See Table S1 for results of all models. We found no significant effect of extraction type on number of loci, average locus depth or standardized depth. Within DNAzol extracted specimens, Net-EtOH had a marginally significantly higher number of loci than Net-Dry specimens (z=2.62, p=0.065). Both Net-EtOH and Vane-Dry specimens had significantly higher levels of average depth than Net-Dry specimens (Net-EtOH: z=4.738, p<0.001; Vane-Dry: z=4.055, p<0.001, Fig S2), with no significant difference in average depth between Net-EtOH and Vane-Dry. We found no significant differences between treatments in standardized depth in DNAzol specimens (Fig. S2e).

*DNA quality*

Of the two DNA quality metrics that significantly affected locus recovery and depth in *B. pensylvanicus* (lower DNA concentration and lower Nanodrop 260/280), only the 260/280 metric was significantly affected by treatment in DNAzol extracted bumblebees (F=8.627, p=0.001). Post-hoc tests revealed that Net-Dry specimens had significantly lower 260/280 ratios than Net-EtOH specimens (p<0.001). DNAzol extracted specimens also had substantially lower 260/230 ratios than Qiagen extracted specimens, indicating presence of salt contaminants. However, this did not significantly affect the amount of recovered loci or depth.

**Table S1.** Complete list of specimens including geographic information, sample year, number of raw reads, number of loci, average locus depth, fragmentation score and DNA weight score. *B.p.= Bombus pensylvanicus, M.t.=Melissodes tepaneca, L.b. = Lasioglossum bardum*

| Species | Sample Treatment | Sample Number | Sample Year | Latitude | Longitude | Raw Reads | Loci | Average Locus Depth | Frag-mentation Score | DNA Weight Score |
|---|---|---|---|---|---|---|---|---|---|---|
| *B.p.* | Net-EtOH | Q004 | 2012 | -96.2391 | 33.1684 | 3819504 | 4010 | 741.43 | low | high |
| *B.p.* | Net-EtOH | Q005 | 2012 | -96.9936 | 32.6078 | 2322205 | 3958 | 539.92 | low | high |
| *B.p.* | Net-EtOH | Q006 | 2012 | -96.9936 | 32.6078 | 3830297 | 3923 | 773.81 | low | high |
| *B.p.* | Net-EtOH | Q007 | 2012 | -96.2391 | 33.1684 | 1965781 | 3952 | 481.49 | high | high |
| *B.p.* | Net-EtOH | Q008 | 2012 | -96.2391 | 33.1684 | 2119833 | 3729 | 508.01 | high | high |
| *B.p.* | Net-EtOH | Q009 | 2012 | -96.2427 | 33.3139 | 3656199 | 3847 | 732.48 | low | low |
| *B.p.* | Net-EtOH | Q010 | 2012 | -96.2391 | 33.1684 | 2538885 | 3911 | 588.04 | high | high |
| *B.p.* | Net-EtOH | Q011 | 2012 | -96.2427 | 33.3139 | 1057320 | 3862 | 278.40 | high | high |
| *B.p.* | Net-EtOH | Q189 | 2012 | -96.2427 | 33.3139 | 3181687 | 9274 | 298.36 | NA | NA |
| *B.p.* | Net-EtOH | Q190 | 2012 | -96.2427 | 33.3139 | 2018871 | 8986 | 189.96 | NA | NA |
| *B.p.* | Net-EtOH | Q191 | 2012 | -96.2427 | 33.3139 | 2794192 | 9165 | 249.41 | NA | NA |
| *B.p.* | Net-EtOH | Q192 | 2012 | -96.2427 | 33.3139 | 202161 | 2253 | 24.01 | NA | NA |
| *B.p.* | Net-EtOH | Q193 | 2012 | -96.2428 | 33.3079 | 6575178 | 10209 | 491.21 | NA | NA |
| *B.p.* | Net-Dry | Q012 | 2014 | -97.3801 | 28.3021 | 1598698 | 4068 | 377.47 | low | high |
| *B.p.* | Net-Dry | Q013 | 2014 | -96.9203 | 28.7114 | 1594666 | 3909 | 369.98 | low | high |
| *B.p.* | Net-Dry | Q014 | 2013 | -97.5934 | 30.3090 | 518726 | 3480 | 133.68 | high | low |
| *B.p.* | Net-Dry | Q015 | 2013 | -97.5934 | 30.3090 | 762893 | 3711 | 194.71 | high | low |
| *B.p.* | Net-Dry | Q016 | 2013 | -97.5934 | 30.3090 | 454471 | 3260 | 128.17 | high | low |
| *B.p.* | Net-Dry | Q017 | 2013 | -97.7813 | 30.2843 | 608152 | 3527 | 156.27 | high | low |
| *B.p.* | Net-Dry | Q018 | 2013 | -96.6189 | 33.1603 | 475683 | 3405 | 124.73 | high | low |
| *B.p.* | Net-Dry | Q019 | 2013 | -96.6189 | 33.1603 | 781802 | 3741 | 184.95 | high | low |
| *B.p.* | Net-Dry | Q194 | 2013 | -97.9573 | 30.0601 | 239261 | 3005 | 28.40 | NA | NA |
| *B.p.* | Net-Dry | Q195 | 2013 | -96.2391 | 33.1684 | 478442 | 5377 | 51.56 | NA | NA |
| *B.p.* | Vane-Dry | Q020 | 2013 | -97.5210 | 30.1913 | 1413663 | 4042 | 329.59 | low | high |
| *B.p.* | Vane-Dry | Q021 | 2013 | -97.6649 | 30.2359 | 877835 | 3631 | 219.19 | low | low |
| *B.p.* | Vane-Dry | Q022 | 2013 | -97.6649 | 30.2359 | 609986 | 3364 | 174.87 | high | high |
| *B.p.* | Vane-Dry | Q023 | 2013 | -97.6649 | 30.2359 | 1344537 | 3989 | 321.88 | high | low |
| *B.p.* | Vane-Dry | Q024 | 2013 | -97.6351 | 30.2862 | 1275172 | 3912 | 309.70 | high | low |
| *B.p.* | Vane-Dry | Q025 | 2013 | -97.2941 | 32.9873 | 1572284 | 3855 | 285.03 | low | high |
| *B.p.* | Vane-Dry | Q026 | 2013 | -97.5210 | 30.1913 | 1352943 | 3913 | 316.94 | high | high |
| *B.p.* | Vane-Dry | Q027 | 2013 | -97.5210 | 30.1913 | 1369062 | 3935 | 332.19 | high | high |
| *M.t.* | Net-EtOH | Q031 | 2013 | -96.8674 | 28.3532 | 2882806 | 13010 | 125.49 | low | high |
| *M.t.* | Net-EtOH | Q032 | 2013 | -96.8674 | 28.3532 | 2037408 | 11366 | 113.15 | low | high |
| *M.t.* | Net-EtOH | Q033 | 2013 | -96.8252 | 28.7512 | 1831761 | 11571 | 109.20 | low | high |
| *M.t.* | Net-EtOH | Q034 | 2013 | -97.2505 | 28.1747 | 1392618 | 11558 | 84.02 | low | high |
| *M.t.* | Net-EtOH | Q035 | 2013 | -96.8607 | 28.4009 | 1711537 | 11148 | 99.71 | low | high |
| *M.t.* | Net-EtOH | Q036 | 2013 | -96.8607 | 28.4009 | 1795418 | 11387 | 98.23 | low | high |
| *M.t.* | Net-EtOH | Q037 | 2013 | -96.8607 | 28.4009 | 1553667 | 11484 | 92.24 | low | high |
| *M.t.* | Net-EtOH | Q038 | 2013 | -96.8607 | 28.4009 | 2596162 | 11647 | 115.38 | low | high |
| *M.t.* | Net-Dry | Q039 | 2013 | -98.2590 | 30.3321 | 794142 | 11786 | 41.00 | high | low |
| *M.t.* | Net-Dry | Q040 | 2013 | -98.2590 | 30.3321 | 6564960 | 13988 | 39.15 | low | high |
| *M.t.* | Net-Dry | Q041 | 2013 | -97.6137 | 30.1978 | 608179 | 10862 | 30.68 | low | low |
| *M.t.* | Net-Dry | Q042 | 2013 | -97.7813 | 30.2843 | 785623 | 11756 | 38.98 | low | low |
| *M.t.* | Net-Dry | Q043 | 2013 | -97.2548 | 28.1898 | 794257 | 10562 | 46.32 | low | high |
| *M.t.* | Net-Dry | Q044 | 2013 | -97.2548 | 28.1898 | 1161278 | 11979 | 61.02 | low | high |
| *M.t.* | Net-Dry | Q045 | 2013 | -97.2360 | 28.1574 | 967106 | 11348 | 51.91 | low | high |
| *M.t.* | Net-Dry | Q046 | 2013 | -96.9101 | 28.3882 | 1221683 | 12729 | 53.69 | low | high |
| *M.t.* | Vane-Gly | Q070 | 2014 | -98.2379 | 30.8896 | 1359432 | 11303 | 87.92 | high | high |
| *M.t.* | Vane-Gly | Q071 | 2014 | -98.2379 | 30.8896 | 1423516 | 10861 | 91.22 | high | high |
| *M.t.* | Vane-Gly | Q072 | 2014 | -98.2379 | 30.8896 | 1079308 | 9900 | 78.25 | low | low |
| *M.t.* | Vane-Gly | Q073 | 2014 | -97.9672 | 30.2126 | 1451509 | 10931 | 94.06 | low | high |
| *M.t.* | Vane-Gly | Q074 | 2014 | -97.9672 | 30.2126 | 1356315 | 10162 | 96.25 | low | high |

| **Species** | **Sample Treatment** | **Sample Number** | **Sample Year** | **Latitude** | **Longitude** | **Raw Reads** | **Loci** | **Average Locus Depth** | **Frag-mentation Score** | **DNA Weight Score** |
|---|---|---|---|---|---|---|---|---|---|---|
| *M.t.* | Vane-Gly | Q075 | 2014 | -97.9227 | 30.4508 | 1390130 | 10501 | 88.52 | low | high |
| *M.t.* | Vane-Gly | Q076 | 2014 | -97.9227 | 30.4508 | 911183 | 8932 | 78.99 | low | high |
| *M.t.* | Vane-Dry | Q047 | 2013 | -97.6991 | 30.2571 | 1948895 | 13552 | 56.90 | high | low |
| *M.t.* | Vane-Dry | Q048 | 2013 | -97.7251 | 30.2821 | 792908 | 11202 | 43.22 | high | high |
| *M.t.* | Vane-Dry | Q049 | 2013 | -97.1730 | 30.0852 | 520044 | 9338 | 36.57 | high | high |
| *M.t.* | Vane-Dry | Q050 | 2013 | -97.6462 | 30.2358 | 960307 | 12294 | 46.43 | low | high |
| *M.t.* | Vane-Dry | Q051 | 2013 | -97.6462 | 30.2358 | 1076373 | 12127 | 54.92 | low | high |
| *M.t.* | Vane-Dry | Q052 | 2013 | -97.6137 | 30.1978 | 1340261 | 12745 | 62.31 | low | low |
| *M.t.* | Vane-Dry | Q053 | 2013 | -97.6137 | 30.1978 | 1013875 | 12508 | 44.14 | low | high |
| *M.t.* | Vane-Dry | Q054 | 2013 | -97.8267 | 30.2063 | 1456210 | 13247 | 63.41 | low | high |
| *M.t.* | Pan | Q062 | 2013 | -96.8748 | 28.3360 | 1249172 | 12711 | 62.48 | high | high |
| *M.t.* | Pan | Q063 | 2013 | -97.3767 | 28.2973 | 861252 | 11275 | 45.41 | high | high |
| *M.t.* | Pan | Q064 | 2013 | -97.2548 | 28.1898 | 978753 | 11735 | 50.63 | low | high |
| *M.t.* | Pan | Q065 | 2013 | -97.2548 | 28.1898 | 1178500 | 13074 | 53.36 | low | high |
| *M.t.* | Pan | Q066 | 2013 | -97.6462 | 30.2358 | 732326 | 10595 | 41.63 | high | high |
| *M.t.* | Pan | Q067 | 2013 | -97.6137 | 30.1978 | 593381 | 10597 | 31.14 | high | low |
| *M.t.* | Pan | Q068 | 2013 | -97.7813 | 30.2843 | 932763 | 10740 | 53.79 | high | low |
| *M.t.* | Pan | Q069 | 2013 | -97.6462 | 30.2358 | 839116 | 11081 | 47.07 | low | high |
| *L.b.* | Net-Dry | Q181 | 2013 | -98.0732 | 30.6226 | 471548 | 14857 | 21.58 | NA | NA |
| *L.b.* | Net-Dry | Q182 | 2013 | -98.0718 | 30.6279 | 335587 | 12594 | 16.09 | NA | NA |
| *L.b.* | Net-Dry | Q183 | 2013 | -98.0764 | 30.6179 | 261216 | 10686 | 14.06 | NA | NA |
| *L.b.* | Net-Dry | Q184 | 2013 | -98.0718 | 30.6279 | 455343 | 15172 | 19.66 | NA | NA |
| *L.b.* | Net-Dry | Q185 | 2013 | -98.0732 | 30.6226 | 384326 | 13406 | 18.90 | NA | NA |
| *L.b.* | Net-Dry | Q186 | 2013 | -98.0718 | 30.6279 | 548322 | 16734 | 22.99 | NA | NA |
| *L.b.* | Net-Dry | Q187 | 2013 | -98.0732 | 30.6226 | 457762 | 14636 | 20.74 | NA | NA |
| *L.b.* | Net-Dry | Q188 | 2013 | -98.0723 | 30.6252 | 234488 | 8823 | 12.92 | NA | NA |
| *L.b.* | Net-Dry | Q198 | 2013 | -98.0732 | 30.6226 | 378054 | 14308 | 19.34 | NA | NA |
| *L.b.* | Vane-Gly | Q157 | 2014 | -98.0638 | 30.0863 | 1390420 | 12904 | 84.33 | NA | NA |
| *L.b.* | Vane-Gly | Q158 | 2014 | -98.0638 | 30.0863 | 1899997 | 12015 | 137.23 | NA | NA |
| *L.b.* | Vane-Gly | Q159 | 2014 | -98.0638 | 30.0863 | 1104924 | 12391 | 48.32 | NA | NA |
| *L.b.* | Vane-Gly | Q160 | 2014 | -97.9227 | 30.4508 | 804757 | 12259 | 56.72 | NA | NA |
| *L.b.* | Vane-Gly | Q161 | 2014 | -98.0638 | 30.0863 | 1040346 | 12667 | 74.78 | NA | NA |
| *L.b.* | Vane-Gly | Q162 | 2014 | -97.9227 | 30.4508 | 918827 | 12242 | 64.55 | NA | NA |
| *L.b.* | Vane-Gly | Q163 | 2014 | -97.9227 | 30.4508 | 785779 | 11466 | 60.69 | NA | NA |
| *L.b.* | Vane-Gly | Q164 | 2014 | -97.9227 | 30.4508 | 709237 | 11347 | 55.07 | NA | NA |
| *L.b.* | Vane-Dry | Q173 | 2013 | -97.9149 | 30.3055 | 1397933 | 22182 | 54.48 | NA | NA |
| *L.b.* | Vane-Dry | Q174 | 2013 | -97.9149 | 30.3055 | 2096407 | 22676 | 41.34 | NA | NA |
| *L.b.* | Vane-Dry | Q175 | 2013 | -97.9149 | 30.3055 | 1883424 | 23300 | 37.85 | NA | NA |
| *L.b.* | Vane-Dry | Q176 | 2013 | -98.0732 | 30.6226 | 399824 | 11622 | 15.25 | NA | NA |
| *L.b.* | Vane-Dry | Q177 | 2013 | -98.0732 | 30.6226 | 1241133 | 15685 | 25.01 | NA | NA |
| *L.b.* | Vane-Dry | Q178 | 2013 | -97.9149 | 30.3055 | 1805004 | 23989 | 53.91 | NA | NA |
| *L.b.* | Vane-Dry | Q179 | 2013 | -98.0732 | 30.6226 | 797406 | 17215 | 22.24 | NA | NA |
| *L.b.* | Vane-Dry | Q180 | 2013 | -98.0732 | 30.6226 | 3027831 | 17603 | 30.23 | NA | NA |
| *L.b.* | Vane-Dry | Q196 | 2013 | -98.0732 | 30.6226 | 698731 | 18649 | 28.36 | NA | NA |
| *L.b.* | Vane-Dry | Q197 | 2013 | -98.0732 | 30.6226 | 958822 | 20931 | 39.32 | NA | NA |
| *L.b.* | Pan | Q165 | 2013 | -98.3311 | 30.4570 | 1423605 | 21220 | 60.81 | NA | NA |
| *L.b.* | Pan | Q166 | 2013 | -98.3311 | 30.4570 | 1120641 | 19936 | 50.88 | NA | NA |
| *L.b.* | Pan | Q167 | 2013 | -98.3311 | 30.4570 | 1206758 | 20644 | 54.14 | NA | NA |
| *L.b.* | Pan | Q168 | 2013 | -98.3311 | 30.4570 | 1401409 | 21513 | 58.73 | NA | NA |
| *L.b.* | Pan | Q169 | 2013 | -97.9572 | 30.0662 | 1135126 | 20905 | 48.33 | NA | NA |
| *L.b.* | Pan | Q170 | 2013 | -97.9572 | 30.0662 | 1028688 | 20415 | 45.50 | NA | NA |
| *L.b.* | Pan | Q171 | 2013 | -97.9572 | 30.0662 | 494027 | 16593 | 23.51 | NA | NA |
| *L.b.* | Pan | Q172 | 2013 | -97.9572 | 30.0662 | 1190399 | 21264 | 50.19 | NA | NA |

**Table S2.** Mean (± SE) DNA quality and ddRAD assembly quality metrics for DNAzol extracted *B. pensylvanicus*.

| Treatment | Nanodrop 260/280 | Nanodrop 260/230 | DNA Concentration (ng/µL) | Mean Polymorphic Loci | Mean Locus Depth |
|---|---|---|---|---|---|
| Net-Dry | 1.93± 0.02 | 0.59± 0.09 | 9.43± 1.05 | 3296.4± 157.2 | 163.9± 43.5 |
| Net-EtOH | 2.06± 0.01 | 0.95± 0.12 | 8.04± 1.15 | 5329.7± 663.5 | 341.8± 49.6 |
| Vane-Dry | 1.99± 0.03 | 0.95± 0.01 | 9.65± 0.76 | 4035.8± 181.2 | 297.5± 20.1 |

**Table S3**. Results from General Linear Mixed Models for *B. pensylvanicus* including sample filters a) one sample, and b-d) 40%, 60%, 80% of *B. pensylvanicus* specimens.

| | | Recovered Loci | | | | Locus Depth | | | | Standardized Depth | | | |
|---|---|---|---|---|---|---|---|---|---|---|---|---|---|
| Minimum Sample Filter | Variable | Estimate | SE | z value | Pr(>\|z\|) | Estimate | SE | z value | Pr(>\|z\|) | Estimate | SE | z value | Pr(>\|z\|) |
| a) One Sample | Vane-Dry (Intercept) | -2.159 | 0.118 | -18.346 | <0.001 | 4.053 | 0.141 | 28.690 | <0.001 | 2.259 | 0.051 | 44.408 | < 0.001 |
| | Net-EtOH | 0.789 | 0.170 | 4.642 | <0.001 | 0.237 | 0.165 | 1.435 | 0.151 | -0.001 | 0.063 | -0.021 | 0.983 |
| | Net-Dry | -1.055 | 0.146 | -7.216 | <0.001 | -0.795 | 0.172 | -4.612 | <0.001 | 0.067 | 0.066 | 1.007 | 0.314 |
| | Qiagen Extracted | 0.717 | 0.138 | 5.178 | <0.001 | -0.374 | 0.234 | -1.599 | 0.110 | 0.173 | 0.054 | 3.199 | 0.001 |
| | DNA concentration | 0.167 | 0.090 | 1.847 | 0.065 | 0.213 | 0.074 | 2.888 | 0.004 | -0.037 | 0.014 | -2.688 | 0.007 |
| | Nanodrop 260/280 | 0.054 | 0.116 | 0.463 | 0.643 | 0.246 | 0.102 | 2.418 | 0.016 | 0.047 | 0.018 | 2.659 | 0.008 |
| | Nanodrop 260/230 | -0.572 | 0.092 | -6.240 | <0.001 | 0.013 | 0.122 | 0.109 | 0.913 | -0.028 | 0.023 | -1.209 | 0.227 |
| | Net-EtOH x Qiagen | -0.119 | 0.257 | -0.462 | 0.644 | 0.375 | 0.174 | 2.156 | 0.031 | -0.139 | 0.041 | -3.397 | 0.001 |
| | Net-Dry x Qiagen | 0.481 | 0.156 | 3.086 | 0.002 | 0.529 | 0.210 | 2.516 | 0.012 | -0.069 | 0.043 | -1.595 | 0.111 |
| b) 40% | Vane-Dry (Intercept) | 1.548 | 0.146 | 10.574 | <0.001 | 5.034 | 0.161 | 31.267 | <0.001 | 3.231 | 0.057 | 56.668 | < 0.001 |
| | Net-EtOH | 0.398 | 0.172 | 2.306 | 0.021 | 0.255 | 0.195 | 1.306 | 0.191 | 0.013 | 0.068 | 0.186 | 0.852 |
| | Net-Dry | -0.405 | 0.182 | -2.227 | 0.026 | -0.794 | 0.235 | -3.384 | 0.001 | 0.062 | 0.072 | 0.858 | 0.391 |
| | Qiagen Extracted | 0.148 | 0.250 | 0.590 | 0.555 | -0.374 | 0.256 | -1.460 | 0.144 | 0.203 | 0.072 | 2.817 | 0.005 |
| | DNA concentration | 0.202 | 0.080 | 2.514 | 0.012 | 0.230 | 0.081 | 2.831 | 0.005 | -0.045 | 0.017 | -2.568 | 0.010 |

| | | Recovered Loci | | | | Locus Depth | | | | Standardized Depth | | | |
|---|---|---|---|---|---|---|---|---|---|---|---|---|---|
| Minimum Sample Filter | Variable | Estimate | SE | z value | Pr(>\|z\|) | Estimate | SE | z value | Pr(>\|z\|) | Estimate | SE | z value | Pr(>\|z\|) |
| | Nanodrop 260/280 | 0.351 | 0.129 | 2.724 | 0.006 | 0.276 | 0.113 | 2.442 | 0.015 | 0.071 | 0.025 | 2.840 | 0.005 |
| | Nanodrop 260/230 | -0.042 | 0.133 | -0.313 | 0.755 | 0.009 | 0.138 | 0.065 | 0.948 | -0.040 | 0.029 | -1.371 | 0.170 |
| | Net-EtOH x Qiagen | -0.388 | 0.233 | -1.664 | 0.096 | 0.344 | 0.246 | 1.398 | 0.162 | -0.177 | 0.055 | -3.227 | 0.001 |
| | Net-Dry x Qiagen | 0.348 | 0.221 | 1.575 | 0.115 | 0.528 | 0.262 | 2.013 | 0.044 | -0.076 | 0.058 | -1.312 | 0.189 |
| c) 60% | Vane-Dry (Intercept) | 2.249 | 0.156 | 14.444 | <0.001 | 5.136 | 0.149 | 34.510 | <0.001 | 3.331 | 0.061 | 54.711 | < 0.001 |
| | Net-EtOH | 0.243 | 0.176 | 1.377 | 0.169 | 0.255 | 0.194 | 1.320 | 0.187 | 0.010 | 0.071 | 0.140 | 0.889 |
| | Net-Dry | -0.310 | 0.195 | -1.592 | 0.111 | -0.801 | 0.197 | -4.060 | <0.001 | 0.054 | 0.075 | 0.717 | 0.473 |
| | Qiagen Extracted | -0.110 | 0.276 | -0.399 | 0.690 | -0.369 | 0.257 | -1.430 | 0.151 | 0.216 | 0.083 | 2.618 | 0.009 |
| | DNA concentration | 0.186 | 0.082 | 2.258 | 0.024 | 0.232 | 0.082 | 2.820 | 0.005 | -0.047 | 0.019 | -2.453 | 0.014 |
| | Nanodrop 260/280 | 0.418 | 0.131 | 3.180 | 0.001 | 0.286 | 0.115 | 2.490 | 0.013 | 0.085 | 0.029 | 2.910 | 0.004 |
| | Nanodrop 260/230 | 0.173 | 0.139 | 1.244 | 0.213 | 0.007 | 0.128 | 0.050 | 0.957 | -0.046 | 0.033 | -1.382 | 0.167 |
| | Net-EtOH x Qiagen | -0.453 | 0.237 | -1.911 | 0.056 | 0.330 | 0.213 | 1.550 | 0.121 | -0.194 | 0.064 | -3.026 | 0.002 |
| | Net-Dry x Qiagen | 0.361 | 0.295 | 1.225 | 0.221 | 0.527 | 0.230 | 2.290 | 0.022 | -0.077 | 0.068 | -1.129 | 0.259 |
| d) 80% | Vane-Dry (Intercept) | 2.558 | 0.147 | 17.388 | <0.001 | 5.224 | 0.176 | 29.757 | <0.001 | 3.419 | 0.064 | 53.737 | < 0.001 |
| | Net-EtOH | 0.157 | 0.180 | 0.876 | 0.381 | 0.258 | 0.210 | 1.228 | 0.219 | 0.013 | 0.075 | 0.178 | 0.858 |
| | Net-Dry | -0.316 | 0.188 | -1.680 | 0.093 | -0.798 | 0.213 | -3.750 | <0.001 | 0.053 | 0.081 | 0.651 | 0.515 |

| | | Recovered Loci | | | | Locus Depth | | | | Standardized Depth | | | |
|---|---|---|---|---|---|---|---|---|---|---|---|---|---|
| Minimum Sample Filter | Variable | Estimate | SE | z value | Pr(>\|z\|) | Estimate | SE | z value | Pr(>\|z\|) | Estimate | SE | z value | Pr(>\|z\|) |
| | Qiagen Extracted | -0.115 | 0.257 | -0.448 | 0.654 | -0.374 | 0.308 | -1.212 | 0.225 | 0.219 | 0.086 | 2.548 | 0.011 |
| | DNA concentration | 0.164 | 0.083 | 1.979 | 0.048 | 0.233 | 0.087 | 2.674 | 0.007 | -0.046 | 0.019 | -2.389 | 0.017 |
| | Nanodrop 260/280 | 0.433 | 0.127 | 3.413 | 0.001 | 0.288 | 0.123 | 2.331 | 0.020 | 0.084 | 0.030 | 2.841 | 0.005 |
| | Nanodrop 260/230 | 0.201 | 0.138 | 1.462 | 0.144 | 0.014 | 0.146 | 0.093 | 0.926 | -0.043 | 0.034 | -1.254 | 0.210 |
| | Net-EtOH x Qiagen | -0.511 | 0.257 | -1.988 | 0.047 | 0.330 | 0.251 | 1.317 | 0.188 | -0.197 | 0.066 | -2.985 | 0.003 |
| | Net-Dry x Qiagen | 0.370 | 0.283 | 1.306 | 0.191 | 0.525 | 0.269 | 1.952 | 0.051 | -0.081 | 0.070 | -1.166 | 0.244 |

**Table S4**. Results from General Linear Mixed Models for *M. tepaneca* including sample filters a) one sample, and b-d) 40%, 60%, 80% of *M. tepaneca* specimens.

| | | Recovered Loci | | | | Locus Depth | | | | Standardized Depth | | | |
|---|---|---|---|---|---|---|---|---|---|---|---|---|---|
| Minimum Sample Filter | Variable | Estimate | SE | z value | Pr(>\|z\|) | Estimate | SE | z value | Pr(>\|z\|) | Estimate | SE | z value | Pr(>\|z\|) |
| a) One Sample | Vane-Dry (Intercept) | -0.389 | 0.040 | -9.830 | <0.001 | 3.001 | 0.080 | 37.630 | <0.001 | 1.572 | 0.075 | 20.953 | < 0.001 |
| | Vane-Gly | -0.531 | 0.056 | -9.474 | <0.001 | 0.179 | 0.134 | 1.340 | 0.180 | 0.161 | 0.125 | 1.293 | 0.196 |
| | Net-EtOH | -0.049 | 0.059 | -0.830 | 0.407 | 0.899 | 0.125 | 7.200 | <0.001 | 0.172 | 0.120 | 1.433 | 0.152 |
| | Net-Dry | 0.030 | 0.058 | 0.510 | 0.610 | -0.041 | 0.128 | -0.320 | 0.746 | -0.298 | 0.110 | -2.722 | 0.007 |
| | Pan | -0.206 | 0.056 | -3.688 | <0.001 | -0.164 | 0.118 | -1.390 | 0.165 | 0.102 | 0.119 | 0.855 | 0.393 |
| | DNA concentration | -0.067 | 0.036 | -1.871 | 0.061 | -0.125 | 0.052 | -2.390 | 0.017 | 0.015 | 0.051 | 0.294 | 0.769 |
| | NanoDrop 260/280 | -0.062 | 0.032 | -1.919 | 0.055 | -0.034 | 0.046 | -0.740 | 0.457 | 0.117 | 0.045 | 2.590 | 0.010 |
| | NanoDrop 260/230 | 0.055 | 0.037 | 1.501 | 0.133 | 0.030 | 0.055 | 0.540 | 0.589 | -0.087 | 0.053 | -1.651 | 0.099 |
| b) 40% | Vane-Dry (Intercept) | 1.691 | 0.057 | 29.442 | <0.001 | 3.340 | 0.091 | 36.550 | <0.001 | 1.913 | 0.080 | 23.929 | <0.001 |
| | Vane-Gly | -0.653 | 0.094 | -6.965 | <0.001 | 0.087 | 0.144 | 0.600 | 0.545 | 0.069 | 0.123 | 0.562 | 0.574 |
| | Net-EtOH | -0.131 | 0.081 | -1.622 | 0.105 | 0.831 | 0.144 | 5.760 | <0.001 | 0.104 | 0.123 | 0.844 | 0.398 |
| | Net-Dry | 0.048 | 0.088 | 0.550 | 0.583 | -0.037 | 0.141 | -0.270 | 0.790 | -0.294 | 0.128 | -2.289 | 0.022 |
| | Pan | -0.146 | 0.094 | -1.561 | 0.119 | -0.180 | 0.133 | -1.360 | 0.175 | 0.086 | 0.120 | 0.720 | 0.471 |
| | DNA concentration | -0.053 | 0.041 | -1.300 | 0.194 | -0.129 | 0.057 | -2.280 | 0.022 | 0.011 | 0.051 | 0.227 | 0.821 |
| | NanoDrop 260/280 | -0.051 | 0.036 | -1.429 | 0.153 | -0.040 | 0.049 | -0.820 | 0.415 | 0.111 | 0.044 | 2.498 | 0.013 |

| | | Recovered Loci | | | | Locus Depth | | | | Standardized Depth | | | |
|---|---|---|---|---|---|---|---|---|---|---|---|---|---|
| Minimum Sample Filter | Variable | Estimate | SE | z value | Pr(>\|z\|) | Estimate | SE | z value | Pr(>\|z\|) | Estimate | SE | z value | Pr(>\|z\|) |
| | NanoDrop 260/230 | 0.037 | 0.043 | 0.859 | 0.390 | 0.025 | 0.059 | 0.420 | 0.673 | -0.092 | 0.053 | -1.729 | 0.084 |
| c) 60% | Vane-Dry (Intercept) | 2.395 | 0.063 | 38.050 | <0.001 | 3.513 | 0.079 | 44.420 | <0.001 | 2.086 | 0.083 | 25.214 | <0.001 |
| | Vane-Gly | -0.700 | 0.110 | -6.350 | <0.001 | 0.063 | 0.133 | 0.470 | 0.638 | 0.045 | 0.139 | 0.323 | 0.747 |
| | Net-EtOH | -0.223 | 0.093 | -2.400 | 0.017 | 0.803 | 0.121 | 6.650 | <0.001 | 0.075 | 0.127 | 0.593 | 0.553 |
| | Net-Dry | 0.053 | 0.103 | 0.520 | 0.603 | -0.014 | 0.125 | -0.110 | 0.909 | -0.271 | 0.132 | -2.060 | 0.039 |
| | Pan | -0.137 | 0.095 | -1.450 | 0.148 | -0.168 | 0.118 | -1.430 | 0.154 | 0.098 | 0.118 | 0.831 | 0.406 |
| | DNA concentration | -0.043 | 0.044 | -0.980 | 0.326 | -0.124 | 0.053 | -2.350 | 0.019 | 0.018 | 0.051 | 0.346 | 0.730 |
| | NanoDrop 260/280 | -0.041 | 0.038 | -1.070 | 0.282 | -0.044 | 0.047 | -0.940 | 0.349 | 0.107 | 0.044 | 2.449 | 0.014 |
| | NanoDrop 260/230 | 0.023 | 0.046 | 0.490 | 0.621 | 0.030 | 0.055 | 0.540 | 0.588 | -0.088 | 0.054 | -1.620 | 0.105 |
| d) 80% | Vane-Dry (Intercept) | 3.222 | 0.083 | 38.830 | <0.001 | 3.740 | 0.086 | 43.660 | <0.001 | 2.312 | 0.088 | 26.256 | <0.001 |
| | Vane-Gly | -0.663 | 0.134 | -4.960 | <0.001 | 0.075 | 0.134 | 0.560 | 0.573 | 0.058 | 0.136 | 0.422 | 0.673 |
| | Net-EtOH | -0.422 | 0.128 | -3.300 | 0.001 | 0.791 | 0.129 | 6.150 | <0.001 | 0.064 | 0.133 | 0.479 | 0.632 |
| | Net-Dry | 0.038 | 0.130 | 0.290 | 0.769 | 0.034 | 0.131 | 0.260 | 0.793 | -0.222 | 0.134 | -1.654 | 0.098 |
| | Pan | -0.072 | 0.121 | -0.600 | 0.550 | -0.146 | 0.122 | -1.200 | 0.230 | 0.120 | 0.124 | 0.971 | 0.331 |
| | DNA concentration | 0.018 | 0.052 | 0.350 | 0.727 | -0.111 | 0.049 | -2.270 | 0.023 | 0.031 | 0.050 | 0.619 | 0.536 |
| | NanoDrop 260/280 | -0.019 | 0.045 | -0.420 | 0.676 | -0.048 | 0.041 | -1.180 | 0.240 | 0.102 | 0.042 | 2.409 | 0.016 |

| | | Recovered Loci | | | | Locus Depth | | | | Standardized Depth | | | |
|---|---|---|---|---|---|---|---|---|---|---|---|---|---|
| Minimum Sample Filter | Variable | Estimate | SE | z value | Pr(>\|z\|) | Estimate | SE | z value | Pr(>\|z\|) | Estimate | SE | z value | Pr(>\|z\|) |
| | NanoDrop 260/230 | 0.018 | 0.055 | 0.330 | 0.741 | 0.051 | 0.051 | 1.000 | 0.317 | -0.066 | 0.052 | -1.256 | 0.209 |

**Table S5**. Results from General Linear Mixed Models for *L. bardum* including sample filters a) one sample, and b-d) 40%, 60%, 80% of *L. bardum* specimens.

| | | Recovered Loci | | | | Locus Depth | | | | Standardized Depth | | | |
|---|---|---|---|---|---|---|---|---|---|---|---|---|---|
| Minimum Sample Filter | Variable | Estimate | SE | z value | Pr(>\|z\|) | Estimate | SE | z value | Pr(>\|z\|) | Estimate | SE | z value | Pr(>\|z\|) |
| a) One Sample | Vane-Dry (Intercept) | -0.218 | 0.039 | -5.630 | <0.001 | 2.945 | 0.115 | 25.509 | <0.001 | 1.211 | 0.058 | 20.723 | <0.001 |
| | Vane-Gly | -1.665 | 0.038 | -43.620 | <0.001 | 0.211 | 0.186 | 1.138 | 0.255 | 0.668 | 0.093 | 7.173 | <0.001 |
| | Net-Dry | -0.945 | 0.039 | -24.030 | <0.001 | -0.590 | 0.187 | -3.152 | 0.002 | 0.835 | 0.105 | 7.926 | <0.001 |
| | Pan | -0.244 | 0.043 | -5.710 | <0.001 | 0.057 | 0.216 | 0.265 | 0.791 | 0.289 | 0.106 | 2.729 | 0.006 |
| | DNA Concentration | 0.268 | 0.033 | 8.000 | <0.001 | 0.083 | 0.060 | 1.377 | 0.169 | -0.176 | 0.035 | -5.061 | <0.001 |
| | NanoDrop 260/280 | -0.063 | 0.036 | -1.740 | 0.081 | -0.038 | 0.092 | -0.413 | 0.679 | -0.170 | 0.051 | -3.311 | 0.001 |
| | NanoDrop 260/230 | 0.166 | 0.038 | 4.320 | <0.001 | 0.171 | 0.137 | 1.247 | 0.212 | 0.367 | 0.072 | 5.118 | <0.001 |
| b) 40% | Vane-Dry (Intercept) | 2.102 | 0.066 | 31.980 | <0.001 | 3.375 | 0.080 | 42.320 | <0.001 | 1.639 | 0.059 | 27.661 | <0.001 |
| | Vane-Gly | -2.089 | 0.114 | -18.290 | <0.001 | 0.252 | 0.125 | 2.010 | 0.045 | 0.698 | 0.097 | 7.179 | <0.001 |
| | Net-Dry | -0.729 | 0.100 | -7.270 | <0.001 | -0.566 | 0.126 | -4.470 | <0.001 | 0.863 | 0.105 | 8.232 | <0.001 |
| | Pan | -0.049 | 0.104 | -0.470 | 0.636 | 0.075 | 0.146 | 0.510 | 0.607 | 0.298 | 0.112 | 2.650 | 0.008 |
| | DNA Concentration | 0.320 | 0.068 | 4.690 | <0.001 | 0.084 | 0.058 | 1.450 | 0.148 | -0.184 | 0.038 | -4.818 | <0.001 |
| | 260/280 | -0.075 | 0.067 | -1.130 | 0.260 | -0.050 | 0.073 | -0.690 | 0.492 | -0.179 | 0.052 | -3.421 | <0.001 |
| | 260/230 | 0.278 | 0.085 | 3.280 | 0.001 | 0.193 | 0.100 | 1.930 | 0.053 | 0.394 | 0.079 | 4.974 | <0.001 |
| c) 60% | Vane-Dry (Intercept) | 2.783 | 0.077 | 36.320 | <0.001 | 3.566 | 0.092 | 38.610 | <0.001 | 1.828 | 0.070 | 26.085 | <0.001 |

| | | Recovered Loci | | | | Locus Depth | | | | Standardized Depth | | | |
|---|---|---|---|---|---|---|---|---|---|---|---|---|---|
| Minimum Sample Filter | Variable | Estimate | SE | z value | Pr(>\|z\|) | Estimate | SE | z value | Pr(>\|z\|) | Estimate | SE | z value | Pr(>\|z\|) |
| | Vane-Gly | -2.089 | 0.117 | -17.900 | <0.001 | 0.257 | 0.149 | 1.730 | 0.084 | 0.703 | 0.111 | 6.303 | <0.001 |
| | Net-Dry | -0.685 | 0.148 | -4.620 | <0.001 | -0.556 | 0.165 | -3.370 | 0.001 | 0.874 | 0.124 | 7.027 | <0.001 |
| | Pan | -0.012 | 0.139 | -0.090 | 0.931 | 0.067 | 0.160 | 0.420 | 0.674 | 0.290 | 0.125 | 2.322 | 0.020 |
| | DNA Concentration | 0.343 | 0.078 | 4.370 | <0.001 | 0.090 | 0.064 | 1.400 | 0.163 | -0.188 | 0.042 | -4.491 | <0.001 |
| | NanoDrop 260/280 | -0.052 | 0.083 | -0.630 | 0.531 | -0.046 | 0.082 | -0.560 | 0.576 | -0.173 | 0.056 | -3.087 | 0.002 |
| | NanoDrop 260/230 | 0.218 | 0.107 | 2.030 | 0.042 | 0.199 | 0.121 | 1.640 | 0.101 | 0.402 | 0.083 | 4.823 | <0.001 |
| d) 80% | Vane-Dry (Intercept) | 3.420 | 0.127 | 26.857 | <0.001 | 3.761 | 0.109 | 34.540 | <0.001 | 2.022 | 0.074 | 27.187 | <0.001 |
| | Vane-Gly | -1.631 | 0.211 | -7.731 | <0.001 | 0.300 | 0.183 | 1.640 | 0.102 | 0.745 | 0.120 | 6.227 | <0.001 |
| | Net-Dry | -0.641 | 0.189 | -3.400 | 0.001 | -0.541 | 0.181 | -3.000 | 0.003 | 0.886 | 0.130 | 6.829 | <0.001 |
| | Pan | 0.169 | 0.223 | 0.758 | 0.448 | 0.046 | 0.211 | 0.220 | 0.826 | 0.270 | 0.140 | 1.925 | 0.054 |
| | DNA Concentration | 0.391 | 0.097 | 4.021 | <0.001 | 0.101 | 0.070 | 1.430 | 0.151 | -0.187 | 0.045 | -4.115 | <0.001 |
| | NanoDrop 260/280 | -0.005 | 0.102 | -0.046 | 0.963 | -0.028 | 0.088 | -0.310 | 0.754 | -0.151 | 0.059 | -2.586 | 0.010 |
| | NanoDrop 260/230 | 0.108 | 0.144 | 0.754 | 0.451 | 0.189 | 0.144 | 1.310 | 0.190 | 0.389 | 0.093 | 4.201 | <0.001 |

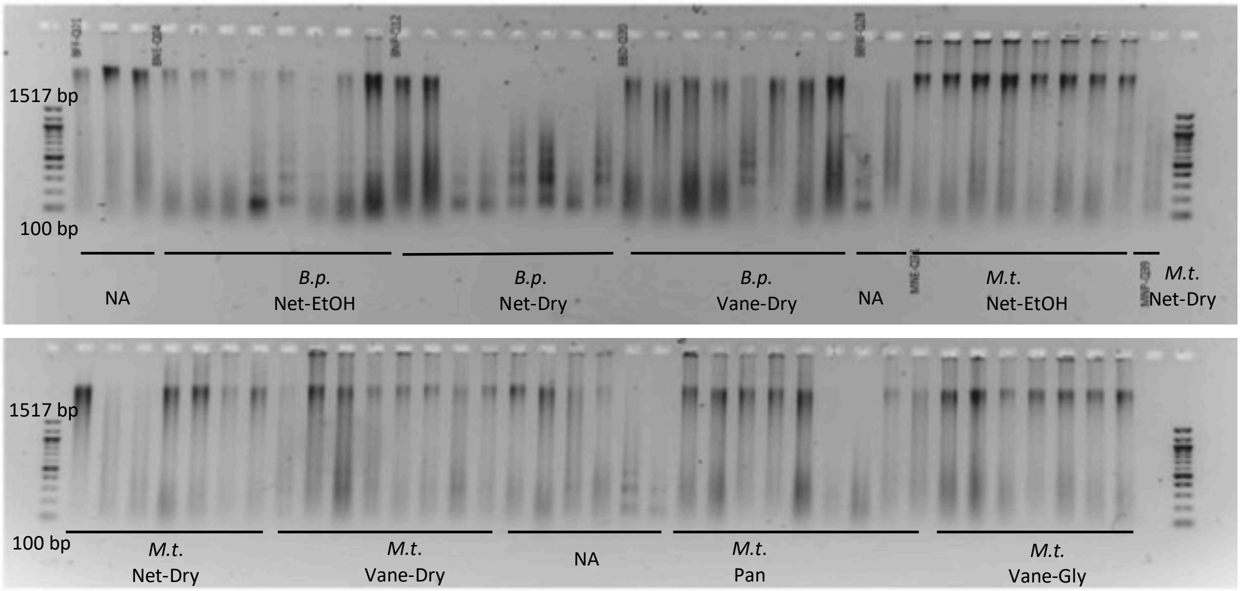


**Fig S1.** Visualization of DNA extractions on a 2% agarose gel showing levels of DNA fragmentation and weight of select samples of Qiagen extracted *B. pensylvanicus* (*B.p.*) and *M. tepaneca* (*M.t.*) by treatment. Samples were orginially given two qualitative DNA quality scores: 1) fragmentation (high = no visible bands or bands ~100-200 bp), and 2) weight (high= dark band above ~1500 bp). Although most samples had “high” fragmentation and “low” weight or vice versa, some samples had bands of both high and low molecular weights, and so would be categorized as both “high” fragmentation and “high” weight (ex. see first two samples in *M.t.* Pan). Ultimately, neither of these measures were significant in initial models, and so were dropped from further analysis. NA indicates samples that were not included in the present study.

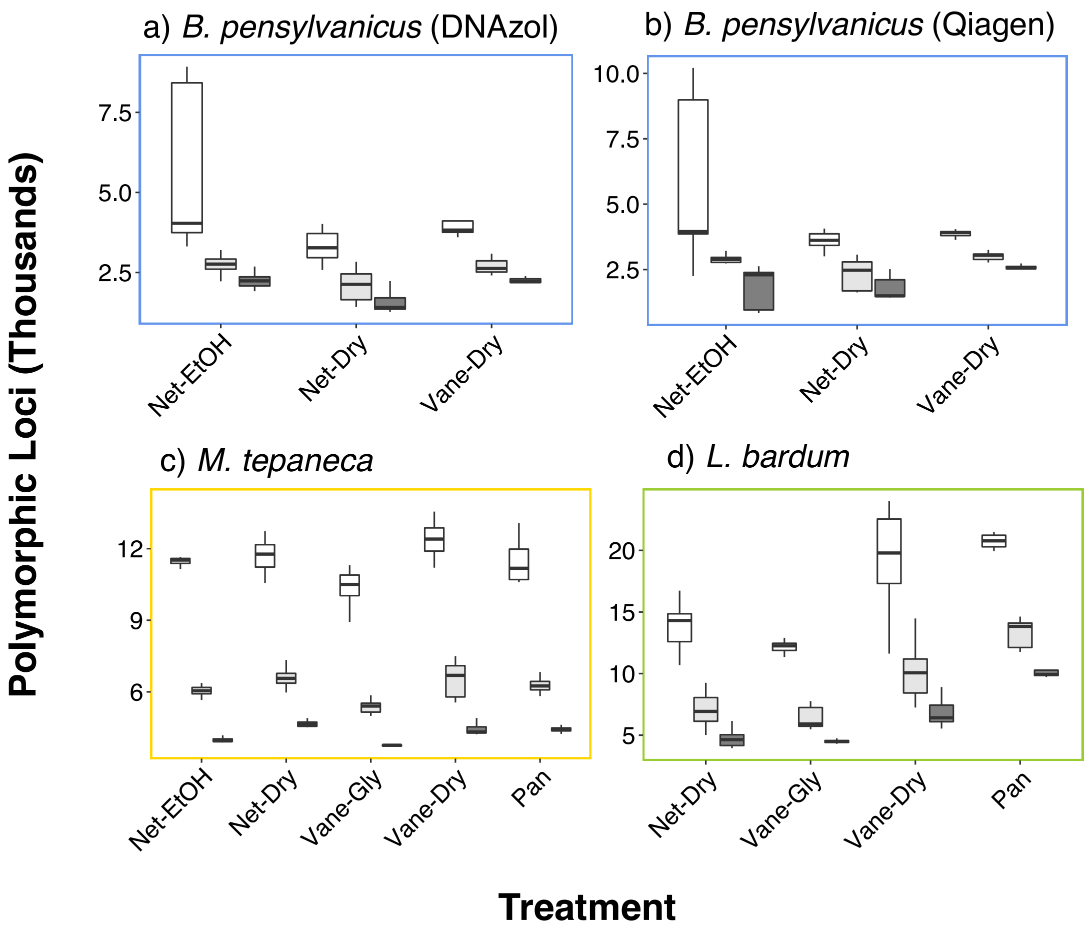


**Fig S2**. Box plots showing numbers of polymorphic loci retained between specimens ("sequence similarity") when grouping by different numbers of individuals. For each species and treatment, groupings are shown from left to right, showing median number of loci and quantile spread when binned by: 1) one individual (left, white), 2) three individuals (middle, light gray), and 3) six individuals (right, dark gray). Sample treatments are coded as in Table 1, plot border color corresponds to species as in Figure 1.

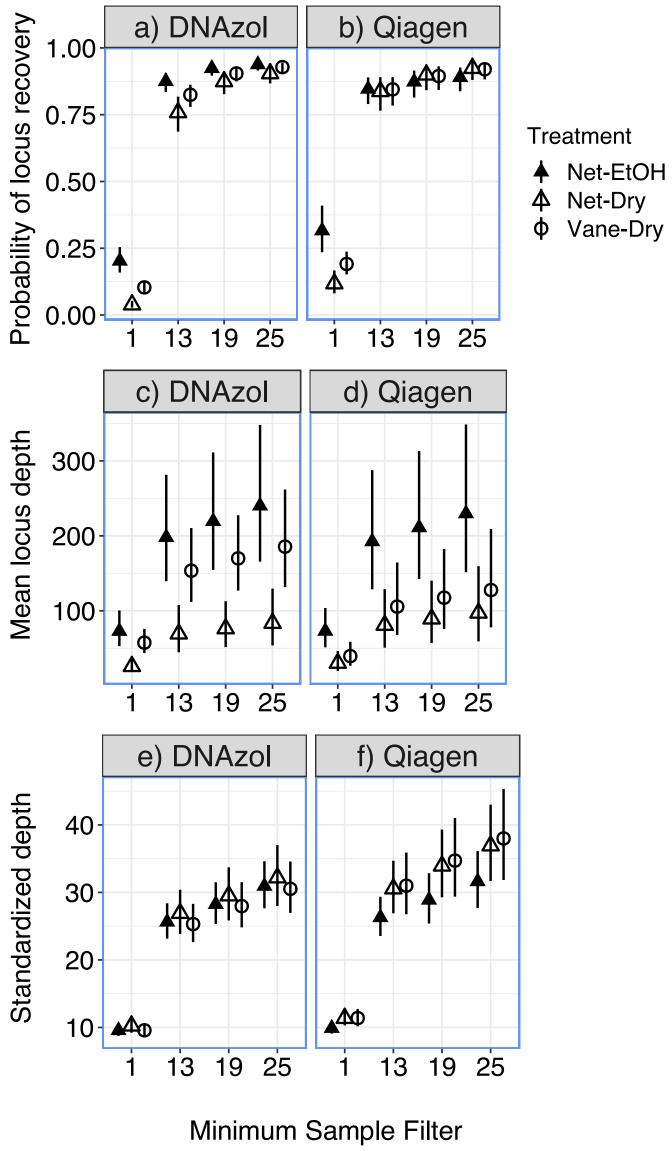


**Fig S3.** Treatment effects on *B. pensylvanicus* ddRAD results, split by extraction method. a,b) Number of loci, measured as scaled probability of a locus occurring in another random sample, c,d) average depth per locus, and e,f) standardized locus depth (average depth per locus per one million reads). All panels show results filtered by a minimum sample of one specimen, and 40%, 60% and 80% of specimens respectively. Bars represent 95% confidence intervals. Sample method-storage treatments are represented by different markers and coded as in Table 1: Vane-Dry (blue vane-dry, pinned; unfilled circles), Net-EtOH (netted, 100% Ethanol; filled triangles) and Net-Dry (netted, pinned; unfilled triangles).

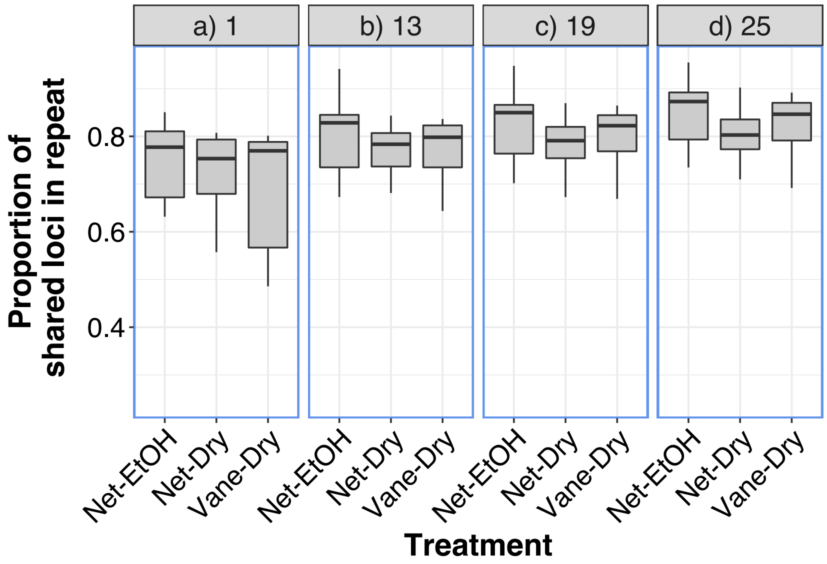


**Fig S4**. Boxplots showing proportion of shared loci between replicate extracted bumblebees (each bumblebee specimen was split in half and extracted using both Qiagen and DNAzol reagants respectively). Plots show proportion of shared loci between extraction types when data is filtered by a minimum of a) 1 specimen, b) 40%, c) 60%, and d) 80% of specimens. There was no significant difference in recovery of matching loci between individuals among the treatments at any sample filter level.

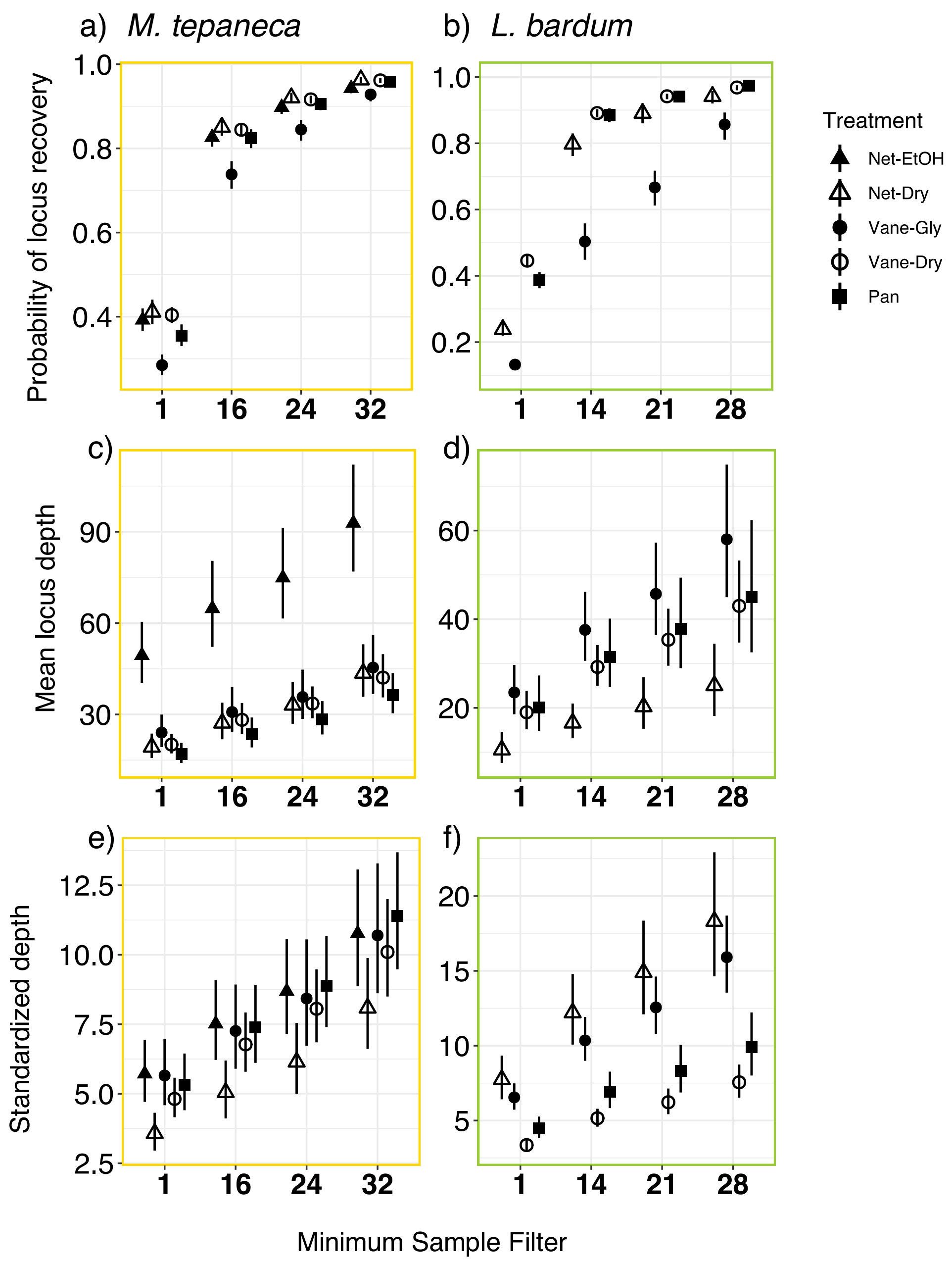


**Fig S5.** Treatment effects on *M. tepaneca* (a, c, e) and *L. bardum* (b, d, f) ddRAD results. a, b) Number of shared loci, measured as scaled probability of a locus occurring in another random sample. c,d) average depth per locus, and e,f) standardized locus depth (average depth per locus per one million reads). All panels show results filtered by a minimum sample of one specimen, and 40%, 60% and 80% of specimens respectively. Bars represent 95% confidence intervals. Sample method-storage treatments are represented by different markers and coded as in Table 1: Net-EtOH (netted, 100% Ethanol; filled triangles), Net-Dry (netted, pinned; unfilled triangles), Vane-Dry (blue vane-dry, pinned; unfilled circles), Vane-Gly (blue vane-glycol, pinned; solid circles), and Pan (pan trap, pinned; solid squares). Panel color corresponds to species as in Figure 1.
